# Unraveling the Developmental Roadmap toward Human Brown Adipose Tissue

**DOI:** 10.1016/j.stemcr.2021.01.013

**Published:** 2021-02-18

**Authors:** Stefania Carobbio, Anne-Claire Guenantin, Myriam Bahri, Sonia Rodriguez-Fdez, Floris Honig, Ioannis Kamzolas, Isabella Samuelson, Kathleen Long, Sherine Awad, Dunja Lukovic, Slaven Erceg, Andrew Bassett, Sasha Mendjan, Ludovic Vallier, Barry S. Rosen, Davide Chiarugi, Antonio Vidal-Puig

**Affiliations:** 1 Wellcome Trust Sanger Institute, Wellcome Trust Genome Campus, Hinxton, UK; 2 Metabolic Research Laboratories, Addenbrooke's Treatment Centre, Institute of Metabolic Science, Addenbrooke's Hospital, University of Cambridge, Cambridge, UK; 3 European Molecular Biology Laboratory, European Bioinformatics Institute, Wellcome Genome Campus, Hinxton, UK; 4 Retinal Degeneration Lab and National Stem Cell Bank-Valencia Node, Research Center Principe Felipe, Valencia, Spain; 5 Stem Cell Therapies for Neurodegenerative Diseases Lab and National Stem Cell Bank – Valencia Node, Research Center Principe Felipe, Valencia, Spain; 6 Institute of Molecular Biotechnology, 1030 Vienna, Austria; 7 Wellcome-MRC Cambridge Stem Cell Institute, Jeffrey Cheah Biomedical Centre, University of Cambridge, Cambridge, UK; 8 Department of Surgery, University of Cambridge, Cambridge, UK; 9 Cambridge University Nanjing Centre of Technology and Innovation, Jiangbei Area, Nanjing, P.R. China

**Keywords:** brown adipose tissue, thermogenesis, BAT progenitors, development, differentiation, human pluripotent stem cells

## Abstract

Increasing brown adipose tissue (BAT) mass and activation is a therapeutic strategy to treat obesity and complications. Obese and diabetic patients possess low amounts of BAT, so an efficient way to expand their mass is necessary. There is limited knowledge about how human BAT develops, differentiates, and is optimally activated. Accessing human BAT is challenging, given its low volume and anatomical dispersion. These constraints make detailed BAT-related developmental and functional mechanistic studies in humans virtually impossible. We have developed and characterized functionally and molecularly a new chemically defined protocol for the differentiation of human pluripotent stem cells (hPSCs) into brown adipocytes (BAs) that overcomes current limitations. This protocol recapitulates step by step the physiological developmental path of human BAT. The BAs obtained express BA and thermogenic markers, are insulin sensitive, and responsive to β-adrenergic stimuli. This new protocol is scalable, enabling the study of human BAs at early stages of development.

## Highlights

• This is a novel, robust and scalable protocol to differentiate hPSCs into BAs
• The protocol recapitulates the different stages of human BAT development
• hPSC-derived BAs show transcriptomic and functional features of bona fide human BAs
• It represents a useful tool for temporal analysis of human BAs differentiation

## Graphical Abstract

**Figure:**
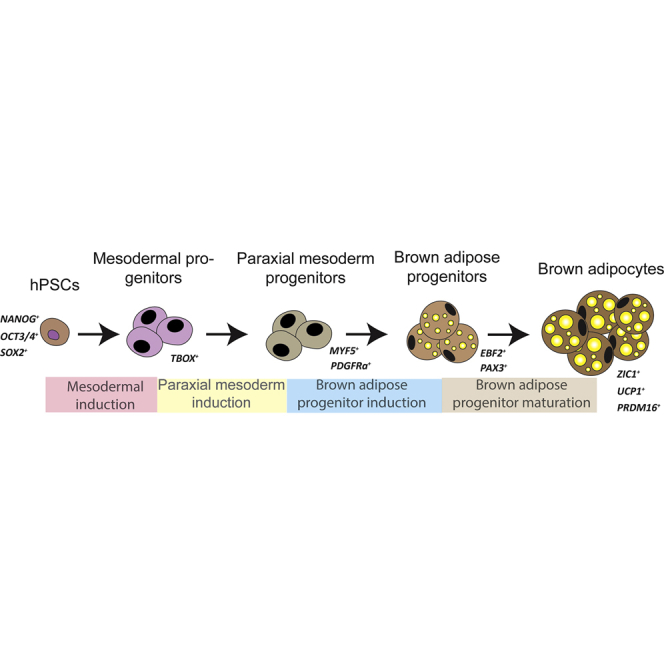

## Teaser

Obese patients possess low amounts of BAT, so an efficient way to expand its mass and activation is necessary to treat obesity and complications. There is limited knowledge about human BAT physiology and accessing it is challenging. To overcome this, Carobbio and colleagues developed a new protocol to differentiate human PSCs into functional brown adipocytes, recapitulating human BAT developmental path.

## Introduction

Obesity and its associated cardiometabolic complications represent a global public health problem. Despite research elucidating the mechanisms controlling energy balance and body weight, the most effective therapy is still bariatric surgery. Given the magnitude of the obesity epidemic, its relevance for cardiometabolic complications, the outcome of infectious diseases, associated human suffering, and economic burden, there is an urgent need for alternative safe, efficient, and cost-effective solutions to combat weight gain and its related comorbidities.

Brown adipose tissue (BAT) is a thermogenic organ able to dissipate energy as heat through regulated mitochondrial uncoupling. BAT thermogenic activity requires activation of a signaling cascade initiated by β-adrenergic stimulation. The thermogenesis critical effector is the mitochondrial uncoupling protein 1 (UCP1), whose function is to allow protons to bypass ATP synthase and dissipate the proton motive force as heat (Nedergaard et al., 2001). In rodents and other small mammals, BAT maintains body temperature. Sustained BAT activation leads to weight loss by promoting energy dissipation resulting in negative energy balance (Badenes et al., 2020; Whittle et al., 2015). From a homeostatic point of view, increasing BAT function would be expected to increase food intake to match energy expenditure (Cannon and Nedergaard, 2004). However, food intake and energy dissipation can be uncoupled. For instance, secretin, a gut hormone secreted in response to food intake, contributes to satiation and BAT stimulation (Li et al., 2018). The dissociation between food intake and energy expenditure provides a therapeutic window leading to net weight loss.

BAT is abundant in small mammals and newborn humans (Cannon and Nedergaard, 2004), both having a high surface/volume ratio that requires increased heat production for thermal homeostasis. Imaging studies conducted in adult humans have shown that BAT is present and functional in most young, lean human adults, particularly when exposed to cold (Chondronikola et al., 2016). Obese and diabetic humans have less BAT (van Marken Lichtenbelt et al., 2009). The lack of BAT in obese patients is partially reversible at low temperatures (Hanssen et al., 2016). This re-appearance of BAT indicates the existence of BAT precursors in adipose tissue that could be differentiated into mature, active adipocytes. In support of this idea, Jespersen et al. (2019) identified widespread amounts of precursor cells and dormant BAT in the human adult perirenal depot. This dormant BAT exhibited a unilocular morphology and a gene expression profile partly overlapping with the subcutaneous white adipose tissue (WAT). Transcriptomics analysis on BAT from obese versus normal weight individuals showed downregulated cellular respiratory pathways in the obese state correlated with a reduction in the thermogenic function of the BAT. Expression of most BAT-specific genes was *not* affected, and isolated adipose progenitors differentiated into thermogenic adipocytes with equal frequency regardless of BMI group (Jespersen et al., 2020). This evidence provides a strong rationale to focus on the differentiation of BAT precursors and the elucidation of the specific stages of human BAT development. As in rodent models, increasing BAT mass and activation improve diabetes and dyslipidemia (Hanssen et al., 2016), which is an attractive and safe therapeutic strategy.

Before BAT activation or differentiation can be considered a feasible target for therapeutic strategies, there are two hurdles to overcome. The first is to understand the mechanisms controlling the mass of human BAT. While detailed information available from murine model systems provides essential insights, there are significant differences between rodent and human BAT regarding thermogenic capacity, marker gene expression, and pharmacological responsiveness (Blondin et al., 2020; Peirce et al., 2014; Ramage et al., 2016). The developmental origin and cell fate decisions determining canonical BAT development in humans are unclear. This lack of fundamental knowledge is partly due to the difficulty in obtaining a sufficient quantity of high-quality human BAT. Getting human BAT requires invasive surgery, poses ethical restrictions in distributing these cells, and limited scalability makes their use difficult (Jespersen et al., 2013).

There are a few well-characterized human immortalized brown adipocyte (BA) cellular models. The degree to which they mimic the *in vivo* situation is unknown (Markussen et al., 2017; Zilberfarb et al., 1997) being suboptimal due to limited accessibility and severe constraints in passaging and scalability.

*In vitro* differentiation of hPSCs is a promising model for studying human BAT development, brown adipogenesis, and mature BA function to overcome these deterrents. Current protocols of hPSC differentiation into BAs fall into two categories. The first relies on derivation of mesenchymal stem cells (MSCs) or embryoid bodies (Nishio et al., 2012; Oka et al., 2019) before applying a chemical adipogenic stimulus (Nishio et al., 2012). The second relies on ectopic overexpression of genes that drive the BA program (Ahfeldt et al., 2012). Both types of protocols bypass key intermediate pathways, making them unsuitable for elucidating the developmental, adipogenic, and thermogenic signaling events leading to BAT formation. This knowledge gap must be addressed for BAT to be useful therapeutically.

Here, we report the development of an upscalable, robust, chemically defined protocol for the differentiation of hPSCs into BAs. This method recapitulates the physiological roadmap of human BAT development, by directing the pluripotent stem cell state toward paraxial mesoderm, then BA progenitors, before finally undergoing adipogenic and functional maturation. This cellular model represents a unique tool to dissect the molecular mechanisms regulating human BAT development and progenitor differentiation.

## Results

### A chemically defined protocol for differentiation of hPSCs into paraxial mesodermal progenitors

**Figure 1:**
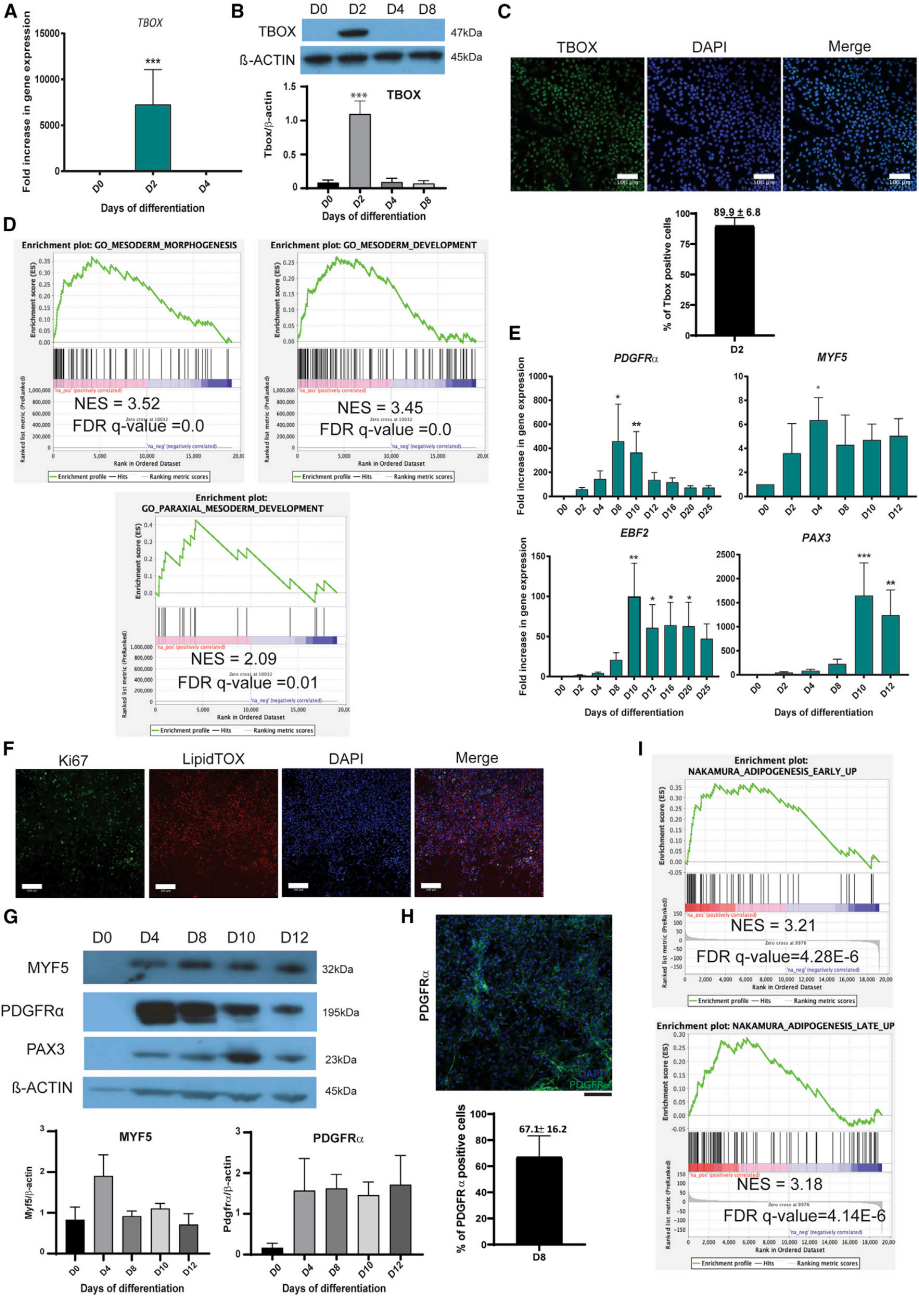
Differentiation of induced pluripotent stem cells into mesoderm and adipose progenitors (A) qRT-PCR analysis of expression of the mesodermal marker *TBOX* in H9-derived mesodermal precursor cells (mean ± SEM arbitrary units [A.U.] relative to D0; n ≥ 3 independent experiments; ^∗∗∗^p < 0.001 relative to D0). *GAPDH* was used as the housekeeping gene (^∗∗∗^p < 0.001 relative to D0). (B) Detection of TBOX in H9-derived mesodermal precursors from D0 to D8 by western blot (WB). β-Actin was used as loading control. Western blot quantification is shown underneath the WB image. (C) TBOX immunostaining of H9-derived mesodermal progenitors at D2 (green). Nuclei were stained with DAPI (blue). Scale bars, 100 μm. TBOX-positive cells were quantified using CellProfiler (SEM ± mean, n = 3 technical replicates). (D) GSEA of H9-derived cells on D4 versus D0 using GSEA (n = 3 independent experiments), using the "mesoderm morphogenesis" GO: 48332, "mesoderm development" GO: 0007498, and "paraxial mesoderm development" GO: 0048339 datasets. (E) qRT-PCR analysis of the expression of the indicated genes from D0 to D12 in H9-derived cells. Data are shown as mean ± SEM A.U. relative to D0; n ≥ 3 experiments; ^∗^p < 0.05, ^∗∗^p < 0.01, ^∗∗∗^p < 0.005 relative to D0. *GAPDH* as housekeeping gene. (F) Ki67 immunostaining of H9-derived adipose progenitors at D12 (green). Nuclei were stained with DAPI (blue). LipidTOX was used to stain the lipid droplets (red). Scale bars, 100 μm. (G) Detection of MYF5, PDGFRα, and PAX3 in differentiating H9 on D0, D4, D8, D10, and D12 by western blot. β-Actin was used as the loading control. Western blot quantification showed underneath the WB image. (H) PDGFRα immunostaining of H9-derived paraxial mesodermal progenitors at D8 (green). Nuclei were stained with DAPI (blue). Scale bars, 100 μm. PDGFRα-positive cells were quantified using CellProfiler (SEM ± mean, n = 3 biological replicates). (I) GSEA of H9-derived adipose progenitor cells using published datasets ("Nakamura adipogenesis early up" and "Nakamura adipogenesis late up"), with early and late adipogenesis transcriptomic signatures on D12 versus D0, compared with human adult adipose stromal cell signature (n = 3 independent experiments).

BAs and skeletal muscle both arise from the paraxial mesoderm, indicating a common origin for these lineages (Seale et al., 2008). We developed a differentiation protocol that first directed pluripotent stem cells toward a mesoderm identity, then to a BAT progenitor state, and finally into mature BAs (Graphical abstract and Figure S1A). To confirm the specificity and reproducibility of this protocol, we validated it in two independent cell lines, the human embryonic stem cell (hESC) line, H9, and the human induced pluripotent stem cell (hiPSC) line, KOLF2-C1 (Figure S2). We confirmed the similarity in the two cell lines' transcriptome at each stage by principal-component analysis (PCA) and clustering analysis. The remarkable similarity in the PCA and heatmap clustering of the two cell lines depended on the development stage rather than the intrinsic differences of origin and genetic background between the cell lines (Figures S1B and S1C). To obtain early mesodermal progenitors from undifferentiated PSCs, we cultured hPSCs for 48 h (day 0 [D0] to D2) in chemically defined medium (CDM) supplemented with insulin, FGF2, and Chiron (GSK3 inhibitor). At D2, we observed the transient upregulation of the mesodermal marker *BRACHYURY* (*TBOX*) as assessed by qPCR, immunoblot, and immunocytochemistry (Figures 1A–1C, S3A, and S3B). The high proportion of TBOX-positive cells indicates the high efficiency of differentiation to mesodermal precursors (Figures 1C and S3B). Over the same period, the expression of pluripotency markers, such as *NANOG*, *SOX2*, and *OCT3/4*, decreased (Figure S2A). Gene set enrichment analysis (GSEA) of the RNA sequencing (RNA-seq) data comparing D4 with D0 confirmed the generation of mesoderm-like progenitors (Figures 1D and S3C).

Paraxial mesoderm arises from the primitive streak (Wymeersch et al., 2016) and gives rise to different cell layers, including the dermomyotome (Sebo et al., 2018) from which BAT and skeletal muscle (Christ and Scaal, 2008) derive. From D2 to D4, we incubated the cells in CDM and added insulin, FGF2, and retinoic acid (RA) to drive the cells to a paraxial mesoderm-like stage (Mendjan et al., 2014). Using this induction cocktail, we obtained progenitor cells expressing mRNA and protein of the paraxial mesoderm markers *MYF5* and *PDGFRα* (Sakurai et al., 2012) (Figures 1E, 1G, and S3A). A total of 67% of cells was positive for PDGFRα (Figures 1H and 3D). GSEA of the RNA-seq data generated from these differentiating cells at D4 compared with D0 confirmed the generation of paraxial mesoderm-like progenitors *in vitro* (Figure 1D). These results confirmed that our protocol promoted paraxial mesodermal precursor development from undifferentiated hPSCs (Wymeersch et al., 2016).

### Differentiation of paraxial mesodermal precursors into BA progenitors

Paraxial mesoderm-like precursors became BA precursor cells upon treatment for 48 h at D4 with insulin, FGF2, Chiron, and LDN-193189 (a BMP inhibitor). Ascorbic acid was used to promote proliferation (Zhang et al., 2016). Following adipocyte precursor formation, the cells were treated with adipogenic induction media between D6 and D8 (DMEM/HAMF12 medium supplemented with triiodothyronine [T3], dexamethasone, 3-isobutyl-1-methylxanthine, biotin, pantothenate, insulin, rosiglitazone, ascorbic acid, and serum). The progenitors proliferated under these conditions, as confirmed at D12 by immunocytochemistry with proliferation marker Ki67 (Figures 1F and S3F). At D12, following 4 days of adipogenic induction (minus ascorbic acid), cells expressed the BA lineage markers *PAX3* and *EBF2* (Figures 1E, 1G, and S3A). GSEA of RNA-seq data comparing D12 and D0 showed a high degree (with a network enrichment score [NES] > 3.1) of similarity in the gene expression signature of these human PSC-derived BAs and primary human stromavascular cells isolated from adipose tissue (Nakamura et al., 2003) (Figures 1H and S3E). These results indicate that the adipogenic induction of hPSC-derived mesodermal precursors drives the emergence of proliferating adipocyte progenitors.

### Generation of human adipocytes expressing classical adipose markers

**Figure 2:**
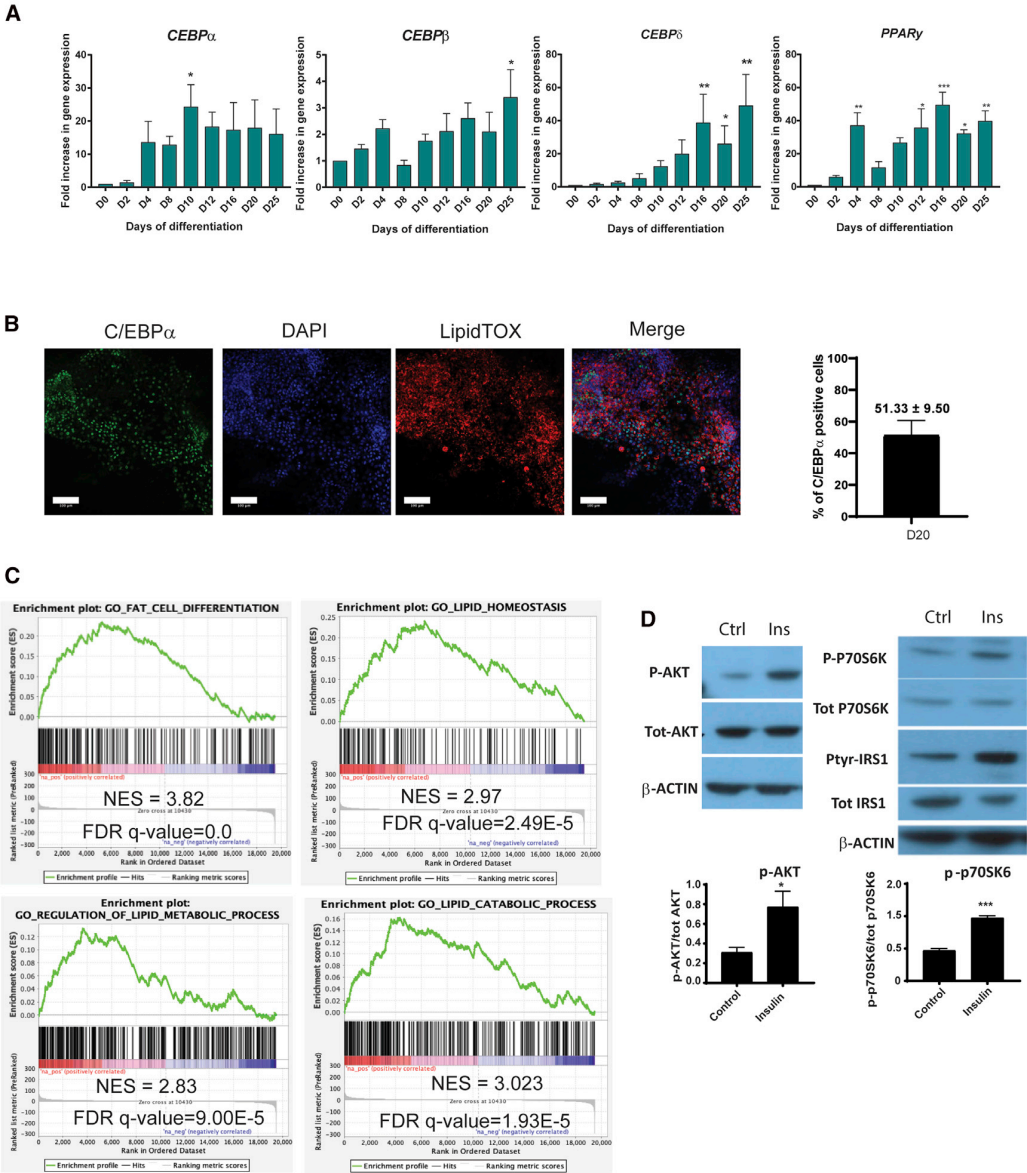
Brown adipocyte progenitor differentiation into adipocytes. (A) Time course analysis of the mRNA abundance of the indicated adipocyte transcription factors during differentiation of H9 (n ≥ 3 experiments; mean ± SEM A.U. ^∗^p < 0.05, ^∗∗^p < 0.01, ^∗∗∗^p < 0.005 relative to D0). *GAPDH* was used as housekeeping gene. (B) Immunodetection of C/EBPα (green) in lipid-containing adipocytes (LipidTOX, red) in H9-derived adipocytes on D20. Nuclei were stained with DAPI. Scale bars, 100 μm. C/EBPα-positive cells were quantified using CellProfiler (SEM ± mean, n = 3 technical replicates). (C) GSEA of H9-derived adipose cells at D25 versus D0 with GO datasets ("fat cell differentiation" GO: 0045444, "lipid homeostasis" GO: 0055088, "regulation of lipid metabolic process" GO: 0006629, and "lipid catabolic process" GO: 0016042) (n = 3 independent experiments). (D) Representative immunoblots showing the phosphorylation of AKT, IRS1, and P70S6K in H9 on D25 treated with 100 nM of insulin for 10 min. β-Actin was used as loading control. The quantification is shown underneath the WB image.

**Figure 3:**
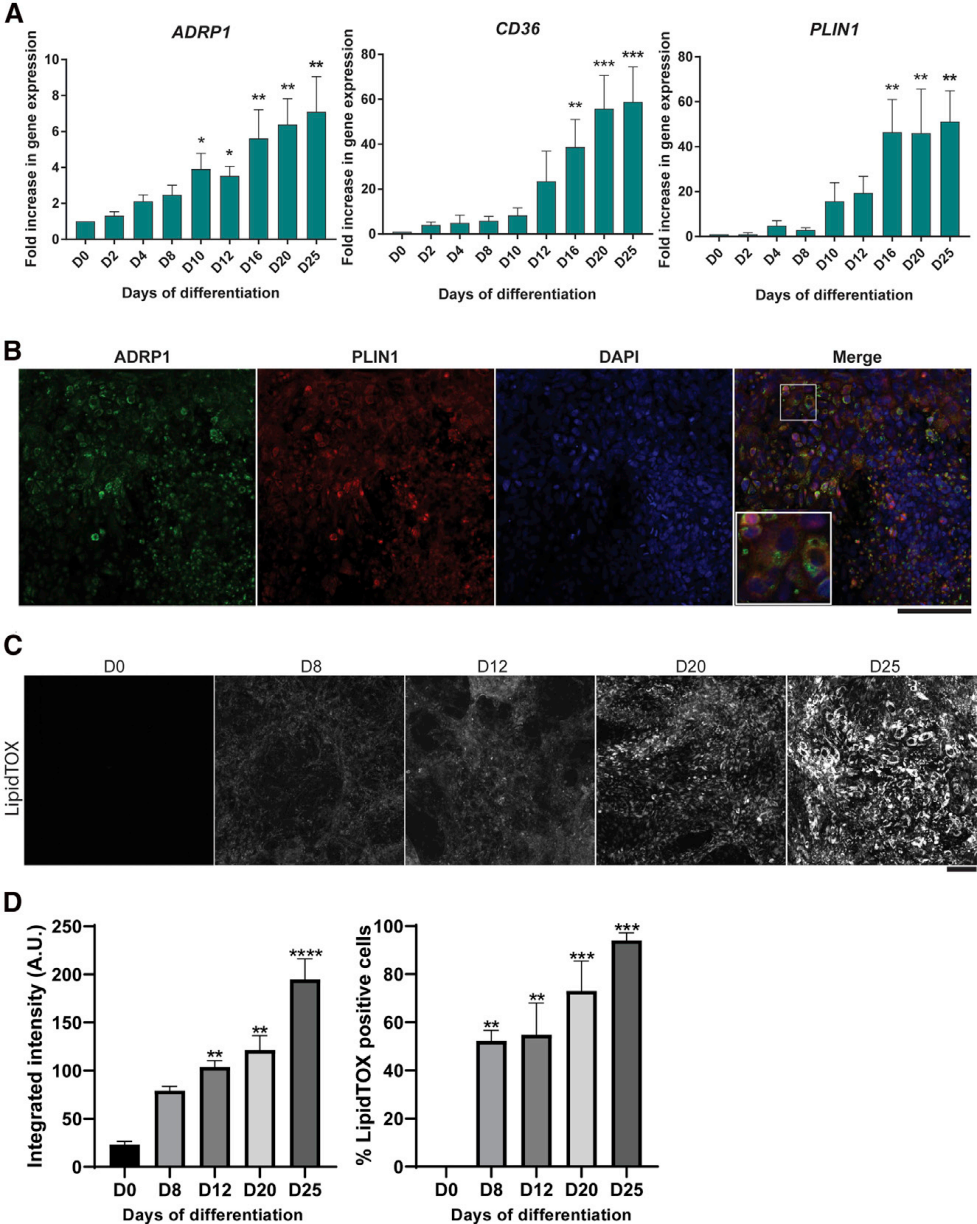
hPSC-derived brown adipocytes accumulate lipids (A) Time course analysis of the mRNA abundance of the indicated critical factors involved in lipid accumulation during adipocyte differentiation in H9 cells (n ≥ 3 experiments; mean ± SEM A.U. ^∗^p < 0.05, ^∗∗^p < 0.01, ^∗∗∗^p < 0.005, relative to D0). *GAPDH* was used as housekeeping gene. (B) Immunodetection of ADRP1 (green) and PLIN1 (red) in H9-derived adipocytes on D25. Nuclei were stained with DAPI (blue). Higher-magnification pictures are shown in the merged image. Scale bar, 100 μm. (C) Representative images of lipid abundance at days 0, 8, 12, 20, and 25 of differentiation detected by LipidTOX staining. Scale bars, 100 μm. (D) Quantification of the LipidTOX signal in the experiments shown in (C). Integrated intensity (left) and percentage of LipidTOX-positive cells (right) throughout differentiation were quantified using CellProfiler. Bar charts represent the mean ± SEM of n = 3 biological replicates (^∗^p < 0.05, ^∗∗^p < 0.01, ^∗∗∗^p < 0.001, ^∗∗∗∗^p < 0.0001 compared with D0, using an ordinary one-way ANOVA test).

After adipogenic induction, the PSC-derived brown precursors were cultured in the presence of T3, dexamethasone, biotin, pantothenate, insulin, rosiglitazone, and oleate from D12 onward. At this stage, cells exhibited increased mRNA expression of adipogenic markers, such as *C/EBPα*, *C/EBPβ*, *C/EBPδ*, and *PPARγ* (Figure 2A), lipid droplet proteins, including *ADRP1* and *PLIN1*, as well as the fatty acid transporter *CD36* (Nassir et al., 2007) (Figure 3A). *C/EBPβ* expression was not transitory, as in white adipogenesis, but sustained, suggesting a more "brown" adipogenic signature. Immunocytochemical detection of C/EBPα, ADRP1, and PLIN1 was observed in cells positive for LipidTOX (Figure 2, Figure 3B, 3B, S3F, and S4A), suggesting that cells showing lipid accumulation were undergoing bona fide adipogenesis. The adipose tissue progenitors positive for C/EBPα represented 51.3% of the cells (Figure 2B). In line with this, LipidTOX intensity levels and the number of LipidTOX-positive cells gradually increased from D0 to D25 of differentiation (Figures 3C, 3D, and S3G). As expected, from the high proportion of lipid-containing cells, GSEA of the PSC-derived adipocytes at D25 showed a strong correlation with the gene ontology (GO) pathways related to "fat differentiation," "lipid homeostasis," "lipid metabolic processes," and "lipid catabolic processes," indicating the development of cells with a coordinated lipid metabolic program (Figures 2C and S4G). Comparable results were observed performing a similar analysis with the PAZ6 human BAs transcriptome at D14 (mature adipocytes) versus D0 (preadipocytes), confirming the similarity of hPSC-derived BAs to human BAs (Figure S5).

The expression of the human BAT markers *KCNK3*, *MTUS1*, and *ITGA10* (Shinoda et al., 2015; Xue et al., 2015) was induced during the differentiation of the hPSC-derived BAs (Figures S6A and S6B). By contrast, the expression of the most commonly used beige markers (Wu et al., 2012) did not show a clear pattern during differentiation, except for *TNFRSF9* and *TMEM26* in H9-derived, but not Kolf2-C1-derived BAs (Figures S6C and S6D).

Assessment of the insulin sensitivity of these human BAs at D25, by measuring AKT phosphorylation at serine 473 following a 10 min stimulation with 100 nM insulin, revealed the increase of AKT phosphorylation in insulin-treated versus -untreated BAs (Figure 2D). Analysis of phosphorylation and total protein expression of other insulin signaling pathway members, i.e., PTyr-IRS1/IRS1 and P-P70S6K/P70S6K (Figure 2D), further validated the insulin sensitivity of the hPSC-derived BAs.

### Human PSC-derived BAs express thermogenic markers

**Figure 4:**
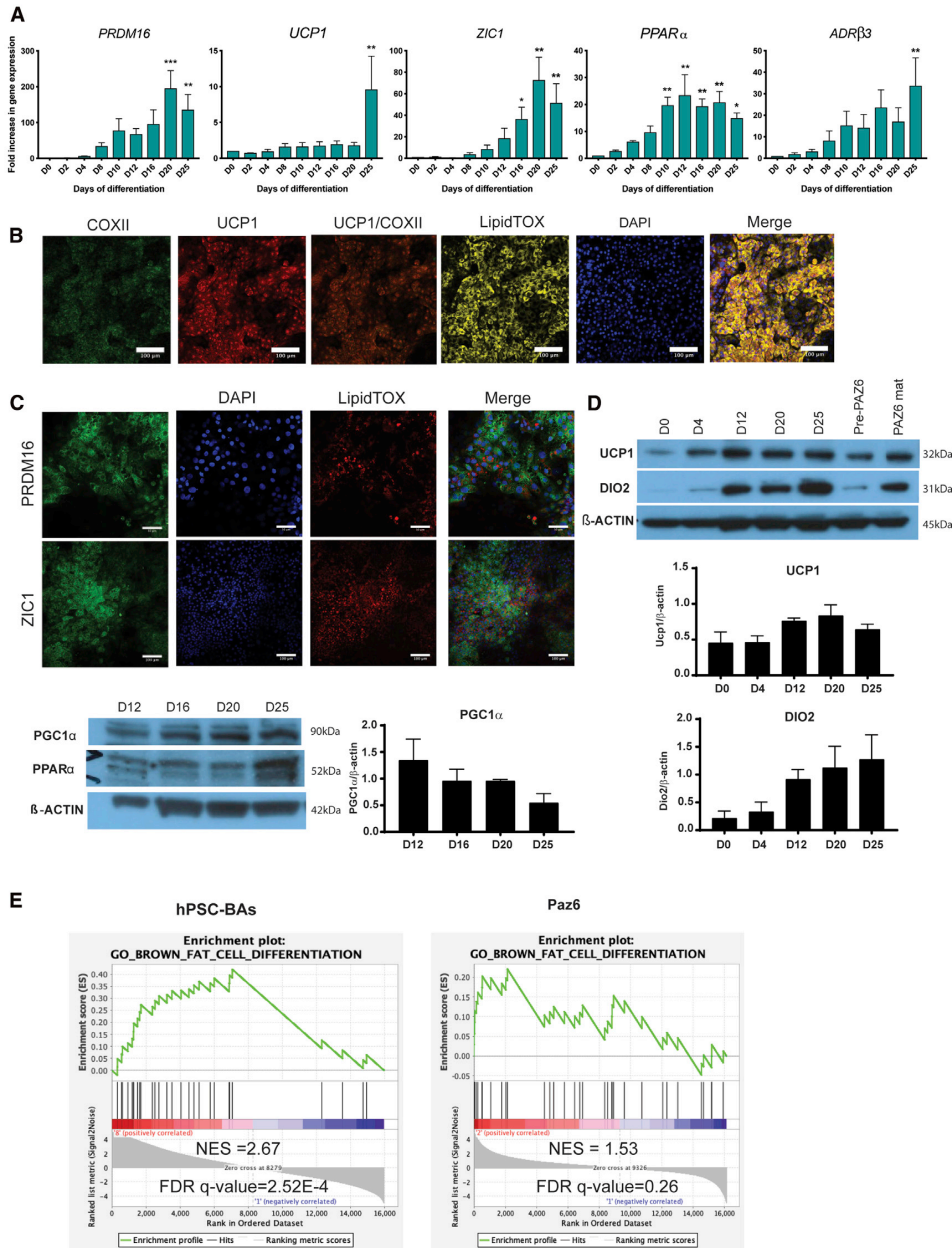
hPSC-derived brown adipocytes are thermogenic (A) Time course analysis of the expression levels of indicated brown adipocyte markers during the differentiation of H9 by qRT-PCR (n ≥ 3 experiments; mean ± SEM A.U. ^∗^p < 0.05 ^∗^p < 0.05, ^∗∗^p < 0.01, ^∗∗∗^p < 0.005 relative to D0). *GAPDH* was used as housekeeping gene. (B) Immunodetection of COXII (green) and UCP1 (red) in lipid-containing (LipidTOX, gray) H9-derived adipocytes on D25. Nuclei were stained with DAPI. Scale bars, 100 μm. (C) Immunodetection of PRDM16 (up) and ZIC1 (down) (green) in lipid-containing (LipidTOX, red) H9-derived adipocytes on D25. Nuclei were stained with DAPI. Scale bars, 100 μm. (D) Representative immunoblots showing the protein levels of UCP1 and DIO2 (top) and PPARα and PGC1α (bottom) in H9-derived brown adipocytes on the indicated day of the differentiation and in the progenitor (pre-PAZ6) and mature stages of the PAZ6 brown adipocyte cell line. β-Actin was used as loading control in both cases. Western blot quantification is shown underneath the image. (E) GSEA of H9-derived adipose cells at D25 versus D0 and PAZ6 human brown adipocytes D14 versus D0 with GO dataset ("brown fat cell differentiation," GO: 0050873) (n = 3 independent experiments).

Sustained induction of *C/EBPβ* is reminiscent of a brown-like adipogenic program (Wang and Seale, 2016). Moreover, hPSC-derived BAs displayed the canonical thermogenic signature of *PRDM16*, *UCP1*, and *ZIC1* (Figure 4A), all classical BA markers (Seale et al., 2007). This signature was induced between D20 and D25 of differentiation and confirmed by immunofluorescence, showing lipid-loaded cells positive for PRDM16 and ZIC1. The same lipid-laden cells co-expressed the mitochondrial markers UCP1 and COXII (Figures 4B, 4C, S4B, and S4C). The expression of UCP1 in human BAs during differentiation was confirmed by western blot. UCP1 was expressed in hPSC-derived adipocytes at D20 and D25 at levels comparable with mature BAs from the PAZ6 BA cell line (Figures 4D and S4C). In addition to UCP1, we detected high levels of PPARα, DIO2, and PGC1α (Figure 4D) at D20 and D25. These cells also expressed *ADRB3* (Figure 4A) encoding the β3-adrenergic receptor. Until recently, the β3-adrenergic receptor was considered the primary β-adrenergic receptor activating mature BAs *in vivo* (Cypess et al., 2015). However, recent studies have reported human BAT activation by ADRB1 or ADRB2 (Blondin et al., 2020). We applied pathway analysis to RNA-seq data and demonstrated that the transcriptome profile of differentiating cells at D25, correlated more strongly with the GO annotation "brown fat cell differentiation" than PAZ6 human BAs (Figures 4E, S5A, and S5B).

**Figure 5:**
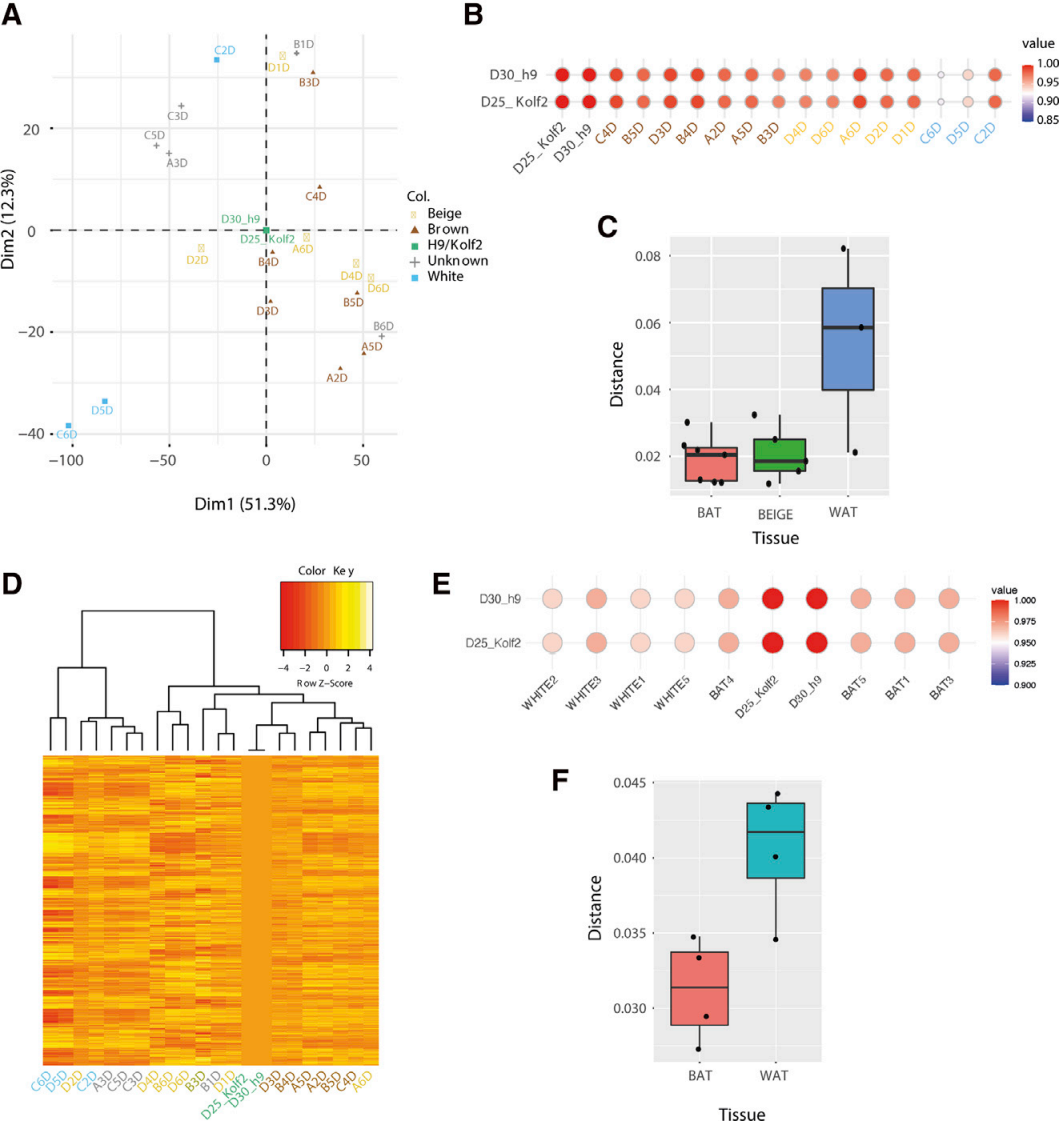
hPSC-derived brown adipocytes are more brown than beige (A and B) Principal-component analysis (A) and correlation matrix (B) of the H9 and KOLF2-C1 stem cell-derived brown adipocytes and different murine BAT clones differentiated *in vitro* (publicly available dataset GSE122780). Dim, dimension. Color code, according to the classification made by (Sun et al., 2020). (C) Euclidean distance between the mature H9 and KOLF2-C1 stem cell-derived brown adipocytes and the murine BAT clones from the dataset used in (A and B). (D) Heatmap and clustering analysis performed on the H9 and KOLF2-C1 stem cell-derived brown adipocytes and different murine BAT clones differentiated *in vitro* (GSE122780). Color code is shown as in (A). (E) Correlation between the H9 and KOLF2-C1 stem cell-derived brown adipocytes and each of the indicated human clones from subcutaneous (WHITE) and supraclavicular (BAT) adipose tissue (publicly available dataset GSE150119). (F) Euclidean distance between the mature H9 and KOLF2-C1 stem cell-derived brown adipocytes and human BAT and WAT clones from the dataset used in (E).

One of the controversies in the field is whether human BAs are brown or beige. Despite the claim that human BAT resembles more murine beige cells than murine BAT (Sharp et al., 2012; Shinoda et al., 2015), recent data indicate that human BAT and murine BAT exhibit similar histology and transcriptional patterns when mice are at thermoneutrality (30°C) (de Jong et al., 2019). Recent studies also indicated that murine BAT is heterogeneous and composed of high and low thermogenic populations with different functional characteristics and expression patterns (Sun et al., 2020). To address interspecies variability, we compared the stem cell-derived BAs with clonally derived cell lines from murine BAT (Figure 5A), finding that they were similar to both clones classified as beige or brown like (Figures 5A–5C), but clustered with those of the brown group (Figure 5D). When compared with immortalized human adipocytes, the stem cell-derived BAs were also closer to the supraclavicular (BAT) than to the subcutaneous (WAT) adipocyte samples (Figures 5E and 5F).

### The transcriptional regulation of human PSC-derived BAs

**Figure 6:**
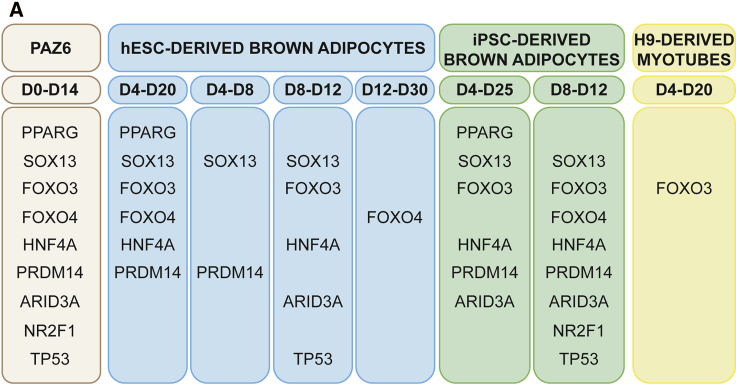
Transcriptional regulators of BAT differentiation (A) Identification of the transcriptional regulators activated in differentiating PAZ6 cells using the VIPER algorithm (left) and their correspondence in the indicated steps of hESC-derived (H9, in blue) and iPSC-derived brown adipocytes (KOLF2-C1, in green). hESC-derived myotubes have been included as a negative control.

We first identified a nine transcriptional regulator signature activated during PAZ6 BAT cell differentiation to strengthen the comparative analysis further. From these nine transcription factors, *PPARG* and *SOX13* are known to regulate brown adipogenesis (Figure 6, left) (Nedergaard et al., 2001). Furthermore, eight of these transcription factors were activated during specific steps of the differentiation of hESCs into BAs, and all nine were activated during iPSC to BA differentiation (Figure 6). Only one of them, *FOXO3*, was activated when the same ESC line was differentiated to skeletal muscle (Figure 6, right) (Wu et al., 2018). Given the similarities in terms of developmental origin between skeletal muscle and BAT, this analysis confirmed our BAT specificity protocol. Altogether, these results outline the specificity of this protocol for human PSC to BA differentiation with all the hallmarks of primary BAs isolated from mice and humans.

### hPSC-derived BAs are functional

**Figure 7:**
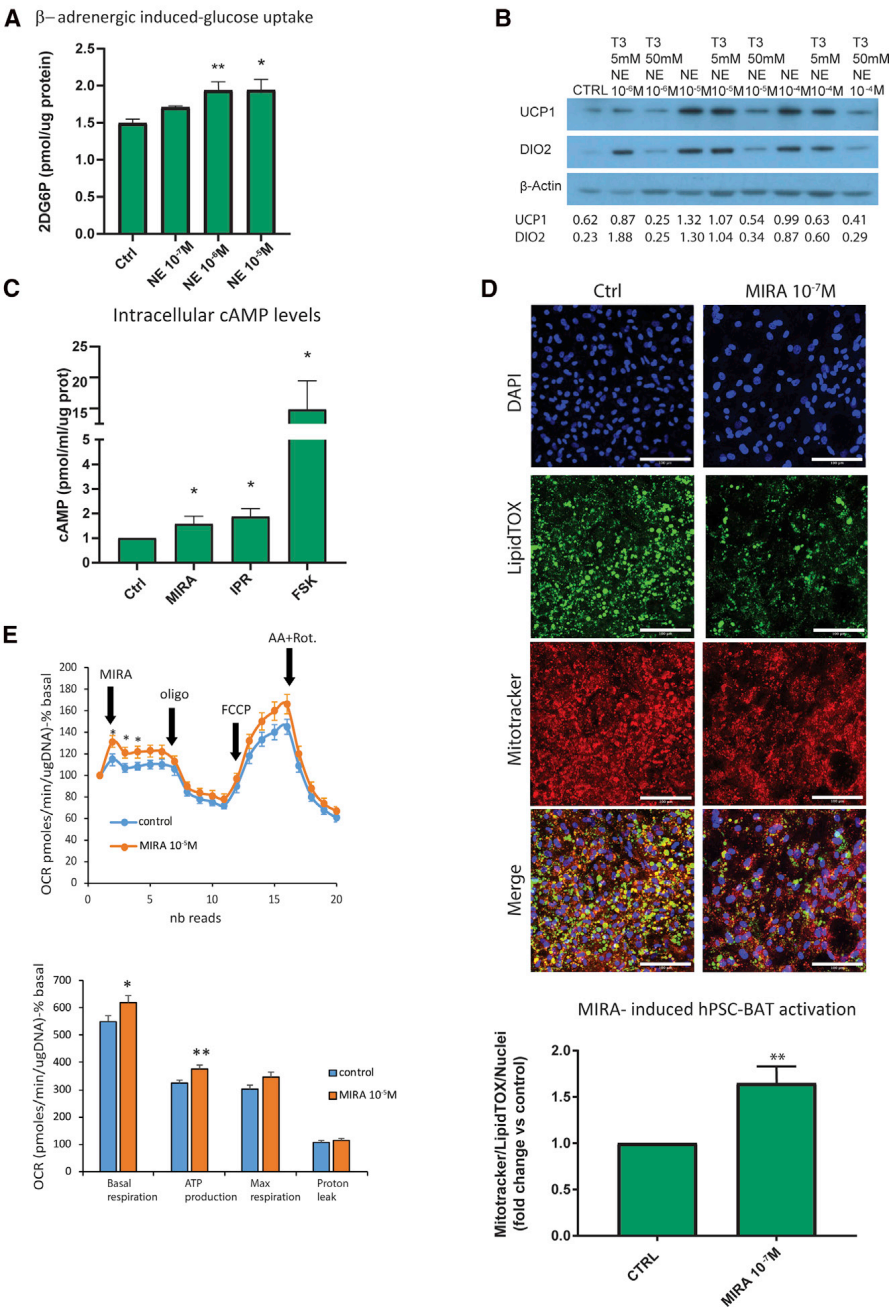
hPSC-derived brown adipocytes respond to β-adrenergic stimuli (A) β-Adrenergic induced glucose uptake was evaluated with the Glucose Uptake Assay Kit (Abcam). At D25, the H9-derived adipocytes cells were treated with different concentrations of norepinephrine (NE) (as indicated in the figure panel). Data are shown as mean ± SEM (n ≥ 3 experiments; ^∗∗^p < 0.005, ^∗^p < 0.05 relative to control; Kruskal-Wallis test). (B) Representative immunoblots showing the levels of UCP1 and DIO2 in H9-derived brown adipocytes at D25 after incubation with the indicated concentrations of NE and T3 for 6 h. β-Actin was used as loading control. Western blot quantification is shown underneath the image. (C) cAMP response to NE, isoproterenol (IPR), and forskolin (FSK), all used at a concentration of 10^−5^ M, was evaluated using the cAMP Parameter Assay Kit (R&D Systems) in fully differentiated H9-derived brown adipocytes. Data are shown as mean ± SEM (n = 3 experiments; ^∗^p < 0.05 relative to control; Kruskal-Wallis test). (D) Upper panel: evaluation of lipid droplet size and activation in a basal state (left panel) and after treatment with MIRA (right panel) using MitoTracker (red) and LipidTOX (green) in H9-derived differentiated adipocytes at D25 using the same confocal settings. Nuclei were stained with DAPI. Scale bars, 100 μm. Lower panel: calculation of the proportion of mitochondria in relation to LipidTOX fluorescence and the number of nuclei under the conditions shown in the upper panel as an indirect measure of the number of activated adipocytes in basal versus treated conditions. Data are shown as mean ± SEM (n = 3 experiments, 5 fields per samples; ^∗∗^p < 0.005 relative to control; Kruskal-Wallis test). (E) Seahorse XF Analyzer profile and quantitative summary of KOLF2-C1-derived BAT stimulated with or without 10^−5^ M MIRA, followed by treatment with 2 μM oligomycin (oligo), 5 μM carbonyl cyanide-4-(trifluoromethoxy)phenylhydrazone (FCCP), and 1 μM antimycin/rotenone (AA + Rot). Data are shown as mean ± SEM (n = 22–25 wells; ^∗^p < 0.05 relative to control; two-tailed Student's t test).

The key functional feature of BAs is their capacity to activate the thermogenic program either by β-adrenergic signaling or with thyroid hormone. Our BA cell system produced a functional human BA that responded to norepinephrine (NE) and mirabegron (MIRA), a β3-adrenergic agonist (Finlin et al., 2018). NE and MIRA stimulation for 2 h increased glucose uptake (Figures 7A, S4D, and S4E). Furthermore, immunoblot analysis showed that the treatment of hPSC-BAs with NE for 6 h increased UCP1 and DIO2 protein expression (Figure 7B). However, overnight treatment with T3 did not further induce UCP1 or DIO2 expression. We tested the ability of different β-adrenergic stimuli to increase intracellular cAMP levels. Firstly, we determined that the general cAMP-stimulating agents isoproterenol and forskolin increased cAMP, indicating that the cells had functional adenylate cyclase as well as phosphodiesterases (Unelius et al., 1993). We demonstrated the responsiveness of cAMP levels to BAT canonical activators by treating cells with MIRA (Figures 7C and S4F). Activation of β3 led to functional downstream readouts of BA activity. MIRA reduced lipid droplet size and increased mitochondrial number (Figure 7D), both known as BAT activation indicators *in vitro* and *in vivo*. Oxygen consumption analysis of human PSC-derived BAs treated with MIRA showed an increase in the basal respiration and ATP production versus untreated controls (Figure 7E). This human BA respiration profile is similar to that of mature murine BAs (Figure S4G). Thus, the hPSC-derived adipocytes obtained from this protocol are fully functional BAs.

### Our hPSC differentiation system is suited for the temporal analysis of human BA differentiation

As the cellular model aimed to recapitulate the developmental steps undergone during brown adipogenesis, we validated the temporal expression of known BAT regulators, markers, and other functionally relevant proteins in humans (Omran and Christian, 2020; Perdikari et al., 2018). This analysis revealed that *C/EBPα*, *C/EBPβ*, and *PRDM16*, for example, are already upregulated in the adipose progenitor state (D8). Another transcriptional regulator, such as *CIDEC* and *PPARγ*, were specifically upregulated later in the differentiation (Figure S7, upper row). Processes such as mitochondrial enrichment and upregulation of fatty acid oxidation enzymes occurred predominantly in the intermediate steps (BAs progenitors/preadipocytes) (Figure S7). This model also helped to identify unexpected comparative biology. For example, when comparing with mouse data, *ADRB3* and the hormone-sensitive lipase (*LIPE*) were found to be already expressed from the first precursor stage, rather than appearing as late-stage differentiation markers (Figure S7). While needing confirmation, by allowing us to parse different developmental stages, our system suggests there may be significant differences between mice and humans in terms of the temporal expression of BA genes. Overall, these results indicate that our stepwise, chemically defined hPSC-to-BA culture system is a potentially powerful tool to gain new insights regarding development and differentiation of human BAs that could prove essential to make BAT a proper therapeutic target in humans.

## Discussion

We have elucidated a step-by-step chemically defined method to differentiate hPSCs into mature BAs. This importance of the method is that it takes pluripotent stem cells through the journey defined by specific developmental stages, closely mimicking the developmental program of BAT *in vivo*. Previous protocols for the differentiation of hPSCs to BAs have used either forced ectopic gene expression of the critical final transcription factors and/or use embryoid body formation and MSC derivation (Ahfeldt et al., 2012; Nishio et al., 2012; Oka et al., 2019). Although successful in generating mature BAT, these cellular models fail to capture the sequence of significant human BAT formation steps, bypassing critical intermediate cellular identities in its development. Our unique vision is that if the ultimate goal is to increase BAT mass in the obese and diabetic population, having access to a clear and detailed characterization of the critical intermediate cell stages connecting PSCs and BAs, is a unique opportunity to rescue the patient's precursors. Targeting intermediate stages with drugs may boost the endogenous capacity for BAT mass formation.

For this vision to succeed, a critical constraint is that the developmental origins of human BAs are not well defined. Most of the available information related to BAT development comes from murine studies (Sanchez-Gurmaches et al., 2016) that have provided the primary evidence that BAs derive from the mesodermal germ layer (Schulz and Tseng, 2013), and more specifically from paraxial mesodermal progenitors (Sebo et al., 2018). Our first question was whether humans might share similar stages for which we optimized a protocol recapitulating the signals that PSCs are exposed to in their journey to become a mature BA. For this, we took human PSCs through the same sequential cell fate specification seen in mice. That this road ultimately develops BA indicates that both species have a high degree of transcriptional similarity, as indicated by the clustering observed between primary human BAs and established cell lines. Thus, we conclude that there is enough similarity in the differentiation pathway between rodents and humans to take advantage of the murine information.

We first induced PSCs toward early mesoderm using a combination of insulin, FGF2, and the GSK3 inhibitor, Chiron, for 2 days. This step was followed by 2 days of insulin, FGF2, and RA, to induce the formation of paraxial mesodermal-like precursors. The rationale behind these specific combinations and sequence of compounds for these particular time frames was to mimic the intrinsic cues that guide the differentiation toward paraxial mesoderm typically observed during the development of the vertebrate embryo *in vivo* (Aulehla and Pourquie, 2010), and accounting for our previous work on PSCs (Mendjan et al., 2014). Our protocol successfully generated *TBOX*^*+*^ early mesoderm progenitors, which subsequently developed into paraxial mesoderm precursors, expressing *MYF5* and *PDGFRα*. Whereas TBOX (Brachyury) is a well-known pan mesodermal marker (Martin and Kimelman, 2010; Mendjan et al., 2014), the induction of *MYF5* and *PDGFRα* confirmed the relevance of paraxial mesoderm genes (Sakurai et al., 2012). The specific cellular identity was confirmed by transcriptomics. While these results provided firm evidence that we had generated paraxial mesoderm, the remaining question was whether these paraxial mesoderm cells would be competent to form BAs.

In the next stage of our protocol, we sought to drive paraxial mesoderm toward adipogenic precursors. We optimized a cocktail, including LDN, a BMP signaling inhibitor, and Chiron, a GSK3 antagonist. LDN and Chiron drive paraxial mesoderm precursors toward presomitic mesoderm-derived lineages, such as muscle and BAT (Carobbio et al., 2013; Chal et al., 2018; Mendjan et al., 2014). This treatment induced the expression of *PAX3* and *EBF2*, two brown cell lineage progenitor markers (Mohsen-Kanson et al., 2014; Sanchez-Gurmaches and Guertin, 2014). GSEA confirmed that the cells generated from the paraxial mesoderm using this protocol were committed to the brown lineage.

At this point, the exposure of the adipogenic precursor cells to a classic adipogenic induction cocktail drove them toward terminally differentiated BAs. As they differentiated, these human PSC-derived BAs increased lipid accumulation and expressed mature adipogenic genes, such as *PLIN1*, and thermogenic genes, including *UCP1*, *DIO2*, and *ADRβ3*. They also expressed transcription factors found in primary mature human BAs, further validating our cellular model.

The next question was whether we were making functionally competent BAs. In response to NE, these cells increased glucose uptake (Inokuma et al., 2005) and induced UCP1 and DIO2 (de Jesus et al., 2001). In response to β3-agonist treatment (Paulo et al., 2018) they had increased cAMP levels and increased basal respiration (Finlin et al., 2018), all typical characteristics of a BA cell.

In summary, here we have developed a step-by-step, robust protocol that recapitulates the developmental stages transforming hPSCs into functional BAs following a rationally designed developmental program road map. This is a unique tool to identify essential key regulatory factors operating at specific stages governing the stage-specific decisions required to guide the PSC toward becoming a fully functional BA. Our protocol avoids the issues caused by the shortcut resulting from engineered ectopic gene expression systems, where changes in expression are driven by the powerful action of the transcription factors introduced. Furthermore, the scalability of the protocol is one of its main assets. It provides a reproducible resource to gain new insights into human BAT formation and physiology suitable for screenings at different developmental stages and the opportunity to study BAs with a specific genotype. Ultimately, we envision that the knowledge generated from studies using this source of inexhaustible human brown fat may enable new safe treatments for obesity and diabetes.

As with every cellular model, it has limitations derived from the use of information coming from non-human organisms, such as rodents as the primary reference, even though human PSC-derived BAs express BAT markers found in human samples (Shinoda et al., 2015; Tran et al., 2020) (Figure 5D, 5F, and S6). Moreover, as with every 2D culture model, signaling interactions with other organs cannot be studied, but it represents the first step to build more complex systems.

## Experimental procedures

### Cell culture

Two hPSCs lines were used. The hESC line H9 (WA09, WiCell, Madison, WI) was maintained in Essential 8 (E8) medium on Vitronectin XF-coated tissue culture-treated dishes. The hiPSC line KOLF2-C1, a subclone of the hiPSC KOLF2 cell line (HPSI0114i-kolf_2, Human Induced Pluripotent Stem Cell Initiative [HipSCi], http://www.hipsci.org) were maintained in TeSR-E8 on Synthemax II-SC Substrate. The human immortalized BA cell line Paz6 was cultured as described previously (Zilberfarb et al., 1997). Mouse adipocytes were cultured as described previously (Garcia-Casarrubios et al., 2016).

### Cell differentiation

For differentiation, pluripotent cells were plated into Matrigel-coated 12-well plates and induced when 70% confluent. At days 0–4, CDM (BSA and insulin-free) (Table S1) was used. From days 6 to 30, complete medium (DMEM-F12 Ham) (see Table S2) was used. For functional analyses, cells were plated onto Matrigel-coated glass-bottomed 96-well plates at D4 of differentiation; cells were detached and plated at single-cell suspension.

### RNA extraction, reverse transcription, and real-time PCR

Primers used for the qPCR are indicated in the primers table found within the Supplemental Information.

### RNA-seq data analysis

Fastq files were processed through a customized pipeline. The adapters were hard clipped before alignment through Cutadapt v.2.3. The alignment was performed using STAR v.2.5 on the GRCh.38. The reads per gene were counted, relying on feature Counts (Subread v.1.6.4). Differential transcriptome analysis was performed using DESeq2, v.1.20.0.

### Analyses of the transcriptional regulators

The inference of the upstream transcriptional regulators was performed with VIPER (Virtual Inference of Protein-activity by Enriched Regulon analysis) (Alvarez et al., 2016).

### GSEA

GSEA (www.broadinstitute.org/GSEA) was carried out on pre-ranked and non-pre-ranked lists of genes. The ranking was computed according to the differential transcriptome analysis mentioned above.

### Immunocytochemistry

For immunocytochemistry, cells were fixed in 4% PFA for 15 min at room temperature and blocked using 3% FFA-free BSA 0.1% Triton X-100 or saponin in PBS. Antibodies and dyes used are described in Table S3. Immunocytochemical quantification was performed with the ICY image analysis software (http://icy.bioimageanalysis.org/).

### Immunoblot

Protein abundance was quantified using a Bio-Rad DC Protein Assay following the manufacturer's instructions. Primary and secondary antibodies used are described in Table S3.

### Insulin sensitivity

Cells were treated with 100 nM of insulin for 10 min, at days 25–30 of differentiation, after an overnight incubation in complete medium without serum. Insulin sensitivity of the cells was assessed by measuring the levels of p-AKT, tot AKT, p-IRS1, tot-IRS1, p-P70S6K, and tot-P70S6K.

### Lipid quantification

Image visualization was performed using Fiji software (Schindelin et al., 2012). Fluorescent images were analyzed using CellProfiler 3.1.9 (McQuin et al., 2018) using custom-built pipelines.

### Seahorse oxygen consumption measurements

Cells were differentiated in a 24-well Seahorse V17 culture plate for 25 days. Before oxygen consumption rate (OCR) assay, complete medium was replaced with Seahorse medium without serum and cytokines. Using a Seahorse XF24 Analyzer, OCR was measured with small-molecule inhibitors added through the injection ports.

### Glucose uptake

NE-induced glucose uptake was assayed according to the Glucose Uptake Assay Kit (Abcam).

### cAMP measurements

The cAMP assay was performed using the cAMP Parameter Assay Kit (R&D Systems, Minneapolis, MN, USA).

### Statistical analysis

All analyses were performed with GraphPad Prism software. After checking the normality of data obtained in each experiment, the appropriate statistical test was applied to calculate significance values between datasets. The following statistical analyses were used to calculate p values: ordinary one-way ANOVA and Kruskal-Wallis. Holm-Sidak's and Dunn's post-hoc tests were performed for multiple comparisons to reduce errors in ordinary one-way ANOVA and Kruskal-Wallis analyses, respectively.

### Data and code availability

The accession number for the RNAseq reported in this paper is and deposited in GEO is GSE158005.

## Author contributions

A.V.-P., S.C., and B.S.R. conceived the original hypothesis. S.C. and A.-C.G. designed and performed *in vitro* experiments, part of the bioinformatics analysis, and wrote the manuscript. M.B., I.S., K.L., and F.H. performed *in vitro* experiments, and discussed and edited the manuscript. D.C. performed the RNA-seq bioinformatics analysis. S.R.-F., I.K., and S.A. performed part of the bioinformatics analysis. S.M. contributed to the design of some of the experiments. A.B. and L.V. contributed by providing advice and some of the cell lines used in this work. D.L. and S.E. participated by guiding some of the *in vitro* experiments. A.V.-P. wrote the manuscript and is the guarantor of this work. All authors approved this publication.

## Supplemental Information

Document S1. Supplemental experimental procedures, Figures S1–S7, and Tables S1–S3

Document S2. Article plus Supplemental information

## Footnotes

Supplemental Information can be found online at https://doi.org/10.1016/j.stemcr.2021.01.013.
