# Supplementary material for: Unraveling the Developmental Roadmap toward Human Brown Adipose Tissue
Source: Stem Cell Reports. 2021 Feb 18;16(3):641–55. doi: 10.1016/j.stemcr.2021.01.013 (PMC7940445; doi:10.1016/j.stemcr.2021.01.013)
Supplement: Document S1. Supplemental experimental procedures, Figures S1–S7, and Tables S1–S3 [file mmc1.pdf]

Stem Cell Reports, Volume *16*

Supplemental Information

Unraveling the Developmental Roadmap toward Human Brown Adipose Tissue

Stefania Carobbio, Anne-Claire Guenantin, Myriam Bahri, Sonia Rodriguez-Fdez, Floris Honig, Ioannis Kamzolas, Isabella Samuelson, Kathleen Long, Sherine Awad, Dunja Lukovic, Slaven Erceg, Andrew Bassett, Sasha Mendjan, Ludovic Vallier, Barry S. Rosen, Davide Chiarugi, and Antonio Vidal-Puig

Supplementary Information

Unravelling the developmental roadmap towards human brown adipose tissue

Stefania Carobbio, Anne-Claire Guenantin, Myriam Bahri, Sonia Rodriguez-Fdez, Floris Honig, Ioannis Kamzolas, Isabella Samuelson, Kathleen Long, Sherine Awad, Dunja Lukovic, Slaven Erceg, Andrew Bassett, Sasha Mendjan, Ludovic Vallier, Barry S. Rosen, Davide Chiarugi and Antonio Vidal-Puig

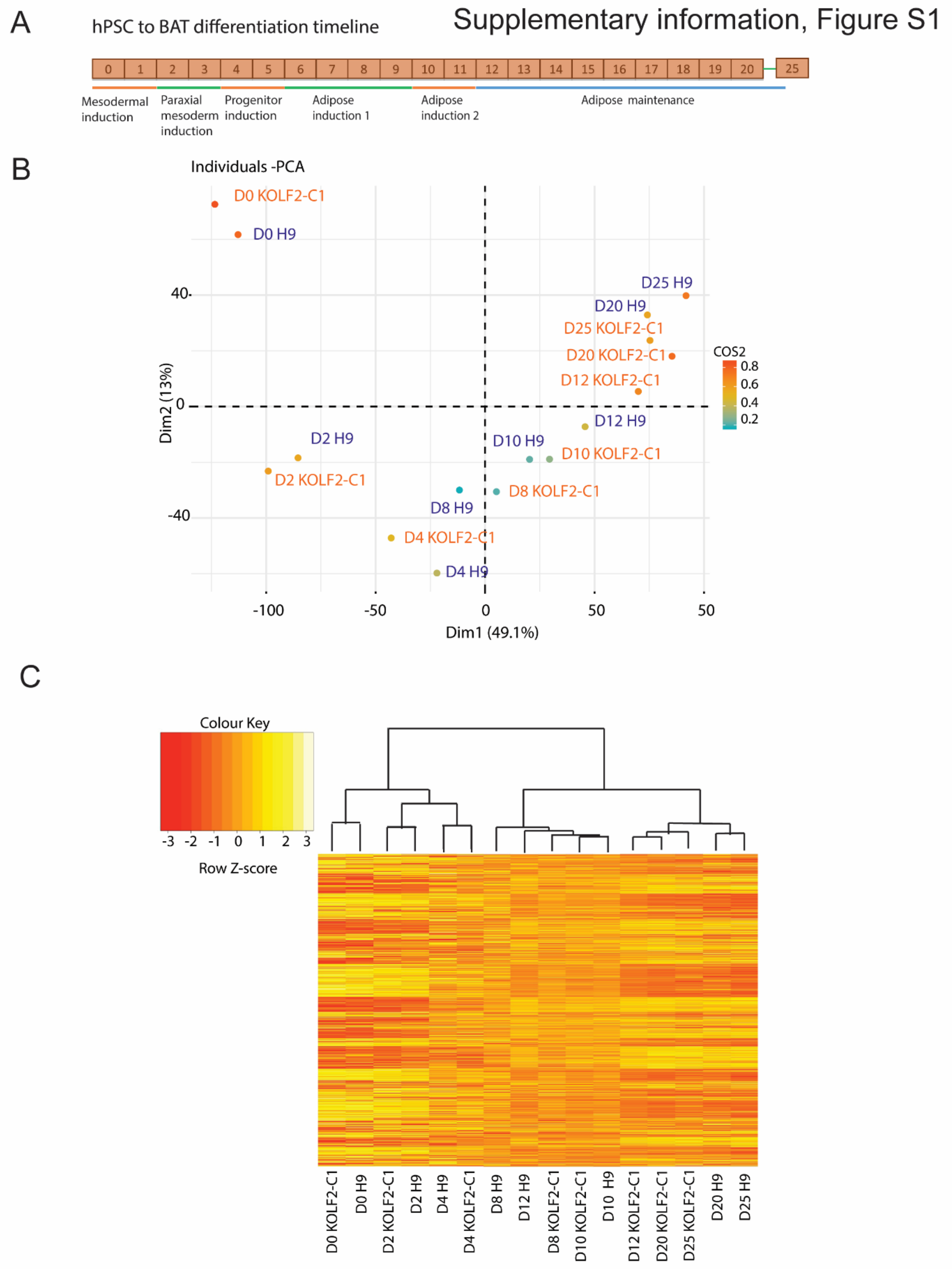

Figure S1. PCA and clustering analysis of H9 and KOLF2-C1 cell lines.

(A) Human PSCs to BAT differentiation protocol timeline.

(B) PCA plot of H9 human ES (here h9) and KOLF2-C1 (here Kolf2) hiPS cell lines differentiation into BAs RNAseq timepoints (D0, D2, D4, D8, D10, D12, D20 and D25). (H9, n=3 and KOLF2-C1 n=5 independent experiments).

(C) Heatmap of clustering analysis of H9 human ES and KOLF2-C1 hiPS cell lines differentiation into BAs. Unsupervised clustering of the D0, D2, D4, D8, D10, D12, D20 and D25 RNAseq timepoints. Upregulated genes are represented in yellow and downregulated clusters in red. (H9, n=3 and KOLF2-C1 n=5 independent experiments).

Supplementary information, Figure S2

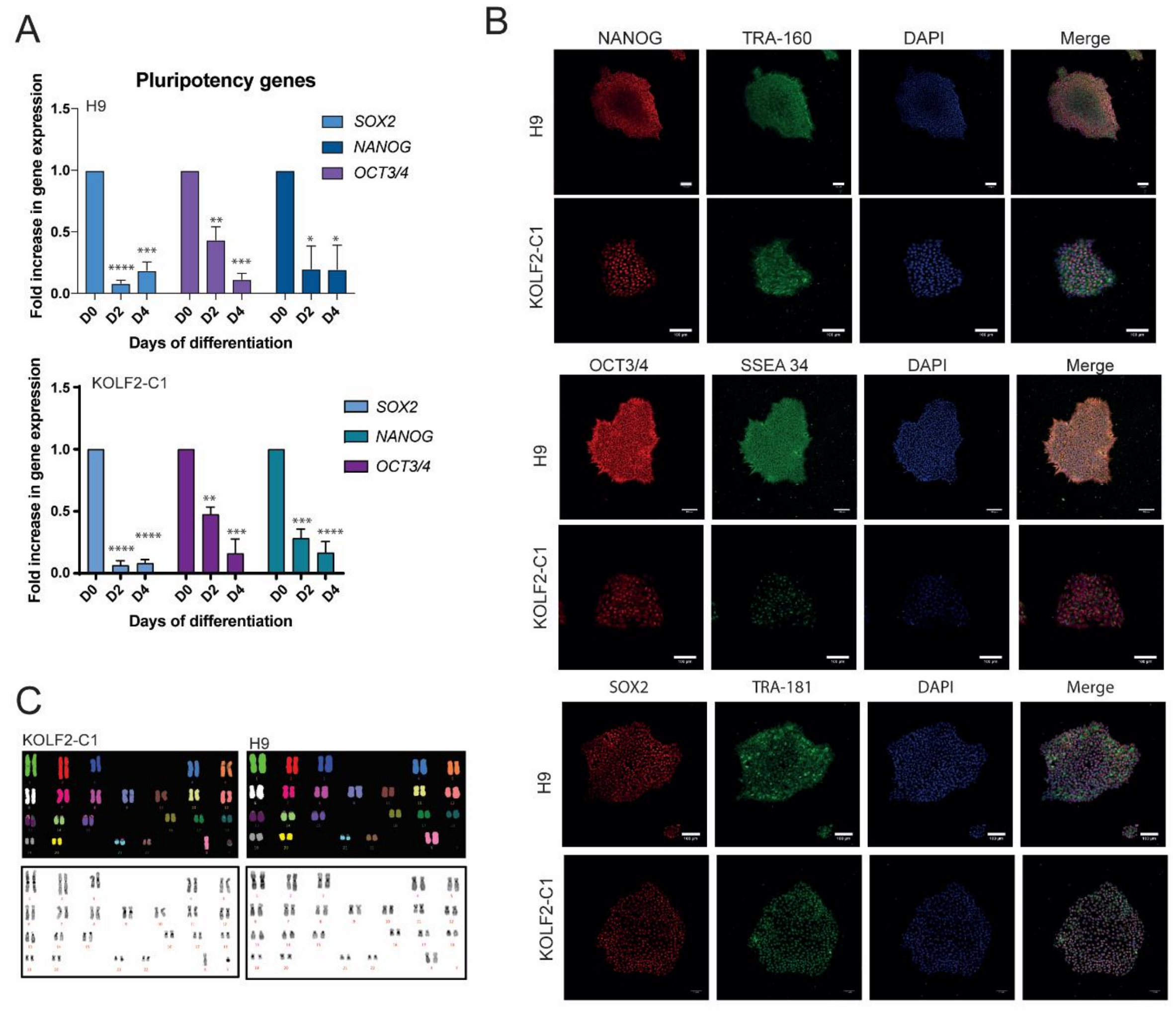

Figure S2. Pluripotency analysis and karyotyping of H9 and KOLF2-C1 cell lines.

(A) RT-qPCR analysis of expression of pluripotency genes *OCT3/4, NANOG* and *SOX2* (mean ± SEM arbitrary units (A.U.) relative to D0; n ≥ 3 independent experiments; **** and ***p<0.0001, **p<0.005, *p<0.05 relative to D0) in H9 (upper panel) and in KOLF2-C1 (lower panel).

(B) Immunodetection of OCT3/4, NANOG and SOX2 (red) co-localised respectively with SSEA 3/4, TRA-1-60 and TRA-1-81 in pluripotent stem cells (H9 and KOLF2-C1) on D0. Nuclei were stained with DAPI. Scale bars: 100 µm

(C) Karyotyping analysis of H9 and KOLF2-C1 cells lines.

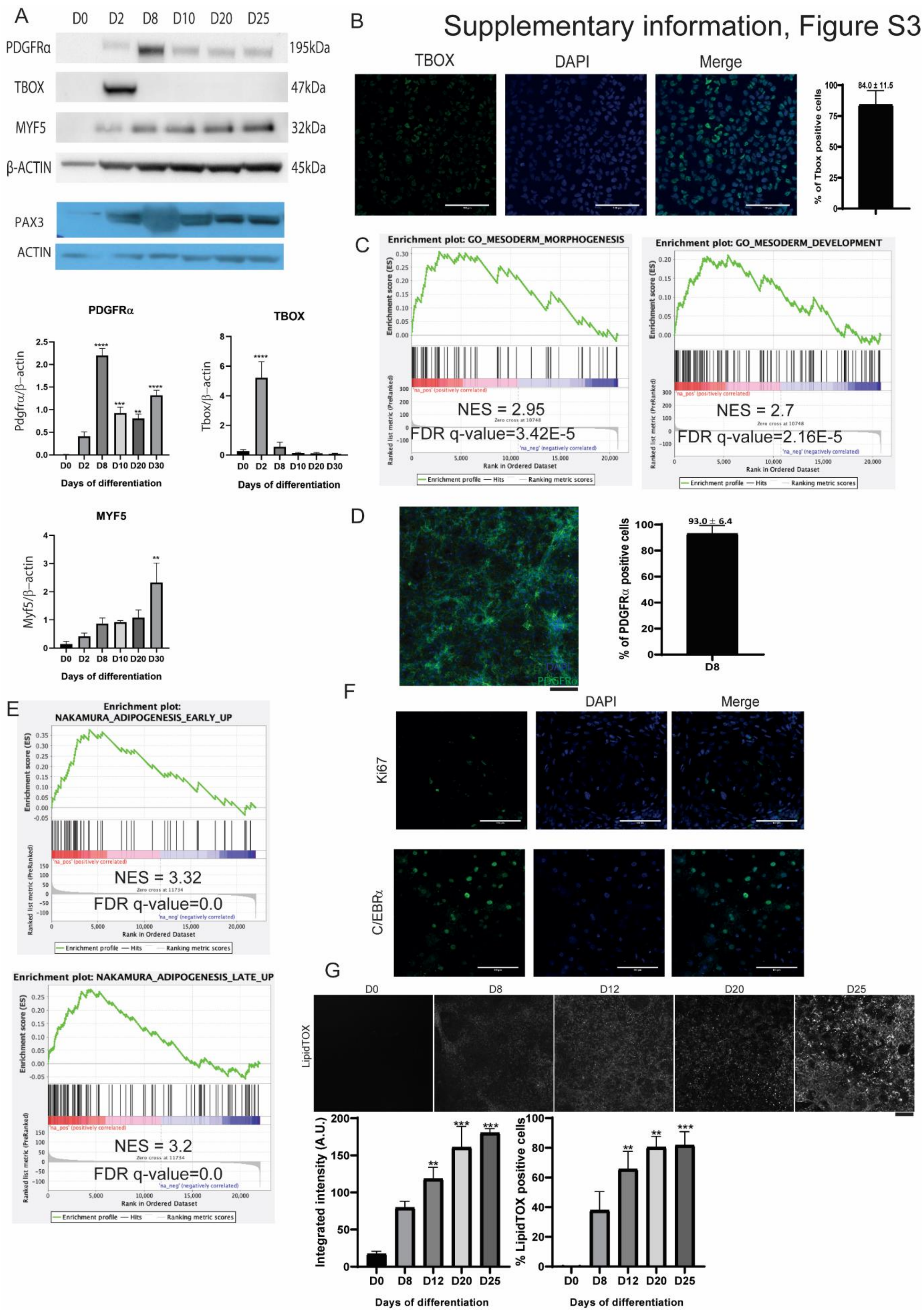

Figure S3. Human iPSC-derived brown adipocytes progenitor molecular characterisation and lipid accumulation during differentiation. Related to

Figures 1, 2 and 3.

(A) Detection of TBOX, MYF5, PAX3 and PGDFRα in KOLF2-C1-derived brown adipocytes on D0, D2, D6, D10, D20 and D25 by western blotting. β-ACTIN was used as loading control. Western blot quantification is shown underneath the WB image. Ordinary one-way ANOVA *p<0.05**p<0.01, ***p<0.001, ****p<0.0001 *vs* D0.

(B)TBOX immunostaining of mesodermal progenitors at D2 (green). Nuclei were stained with DAPI. Bars: 100 µm. TBOX positive cells were quantified using CellProfiler (mean + SEM, n=3 technical replicates). Scale bars: 100 µm.

(C) Gene Set Enrichment Analysis of KOLF2-C1-derived cells on D4 *vs* D0 using GSEA (n=5 independent experiments) using the “mesoderm morphogenesis” GO:48332, “mesoderm development” GO:0007498 datasets.

(D) Immunostaining PDGFRα in KOLF2-C1-derived brown adipocytes on D8, PDGFRα positive cells were quantified using CellProfiler (mean + SEM, n=3 biological replicates). Scale bars: 100 µm.

(E) Gene Set Enrichment Analysis of KOLF2-C1-derived adipose progenitors cells using published datasets (“Nakamura adipogenesis early up” and “Nakamura adipogenesis late up”), with early and late adipogenesis transcriptomic signatures on D12 *vs* D0, compared to human adult adipose stromal cell signature (n=5 independent experiments).

(F) Ki67 immunostaining of adipose progenitors at D12 (green). C/EBPα immunostaining of adipocytes at D25 (green). Nuclei were stained with DAPI (blue). Scale bars: 100 µm.

(G) Representative images of lipid abundance at day 0, 8, 12, 20 and 25 of differentiation detected by LipidTOX staining. LipidTOX immunodetection quantification reveals increased levels of integrated intensity (left) and percentage of

LipidTOX positive cells (right) over the course of differentiation. Bar chart of integrated intensities represents mean ± SEM measured in the cytoplasm of individual cells. Other bar charts represent the mean ± SEM of n=3 biological replicates (*$p<0.05$, **$p<0.01$, ***$p<0.001$ and ****$p<0.0001$ compared to D0, Ordinary one-way ANOVA). Scale bars: 100 µm.

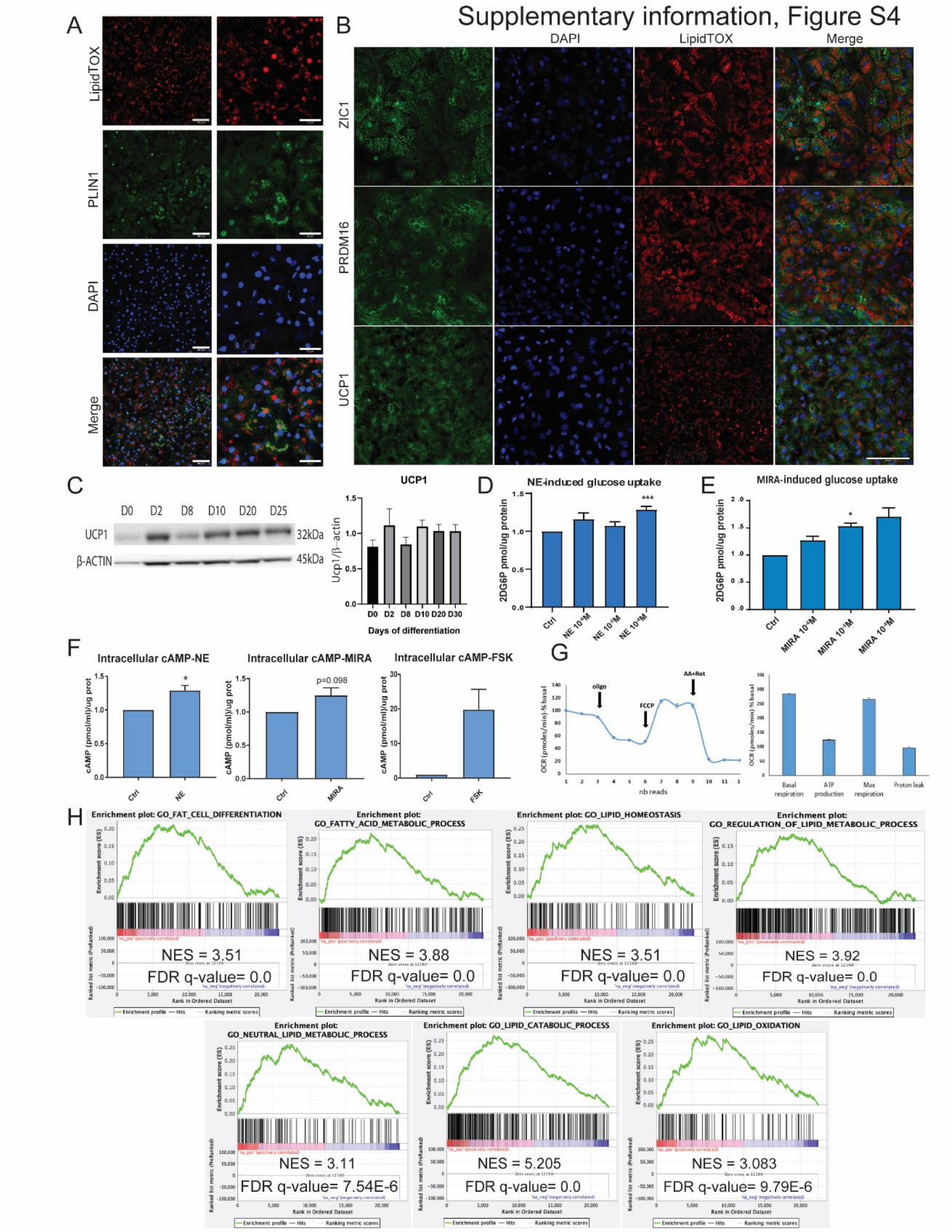

Figure S4. Human iPS-derived brown adipocyte molecular characterisation and metabolic phenotyping. Related to Figure 2, 3, 4 and 7.

(A) Immunodetection of PLIN1 (green) in KOLF2-C1-derived lipid-containing adipocytes (LipidTOX-red) on D25. Nuclei were stained with DAPI. Scale bars 100 µm.

(B) Immunodetection of ZIC1, PRDM16 and UCP1 (green) in lipid-containing (LipidTOX-red) adipocytes (KOLF2-C1) on D25. Nuclei were stained with DAPI. Scale bars 100 µm.

(C) Detection of UCP1 in KOLF2-C1-derived brown adipocytes on D4, D8, D10, D20 and D25 by western blotting. β-ACTIN was used as loading control. Western blot quantification is shown underneath the WB image.

(D) NE- induced glucose uptake was evaluated with the Glucose uptake assay kit (Abcam). At D25, the cells were treated with different concentrations of NE (as indicated in the figure panel) (mean ± SEM $n \geq 3$ experiments; ***$p<0.001$ and * relative to control; Kruskal-Wallis test).

(E) MIRA-induced glucose uptake was evaluated with the Glucose uptake assay kit (Abcam). At D25, the cells were treated with different concentrations of MIRA (as indicated in the figure panel) (mean ± SEM n= 3 wells; *$p<0.05$ relative to control; Kruskal-Wallis test).

(F) cAMP levels in response to NE, MIRA and FSK treatment, all used at a concentration of 10^{-5}M, (mean ± SEM n = 2-4 experiments, *$p<0.05$ relative to control, Kruskal-Wallis test).

(G) Seahorse XF analyser profile and quantitative summary of mouse brown adipocytes following by treatment with 1µM oligomycin (oligo), 0.9µM FCCP and 1µM antimycin/rotenone (AA+Rot). (mean ± SEM n = 10 wells.

(H) Gene Set Enrichment Analysis of KOLF2-C1-derived adipose cells at D25 *vs* D0 with GO datasets (“fat cell differentiation” GO:0045444, “fatty acid metabolic process” GO:0006631, “lipid homeostasis” GO:0055088, “regulation of lipid metabolic process”.

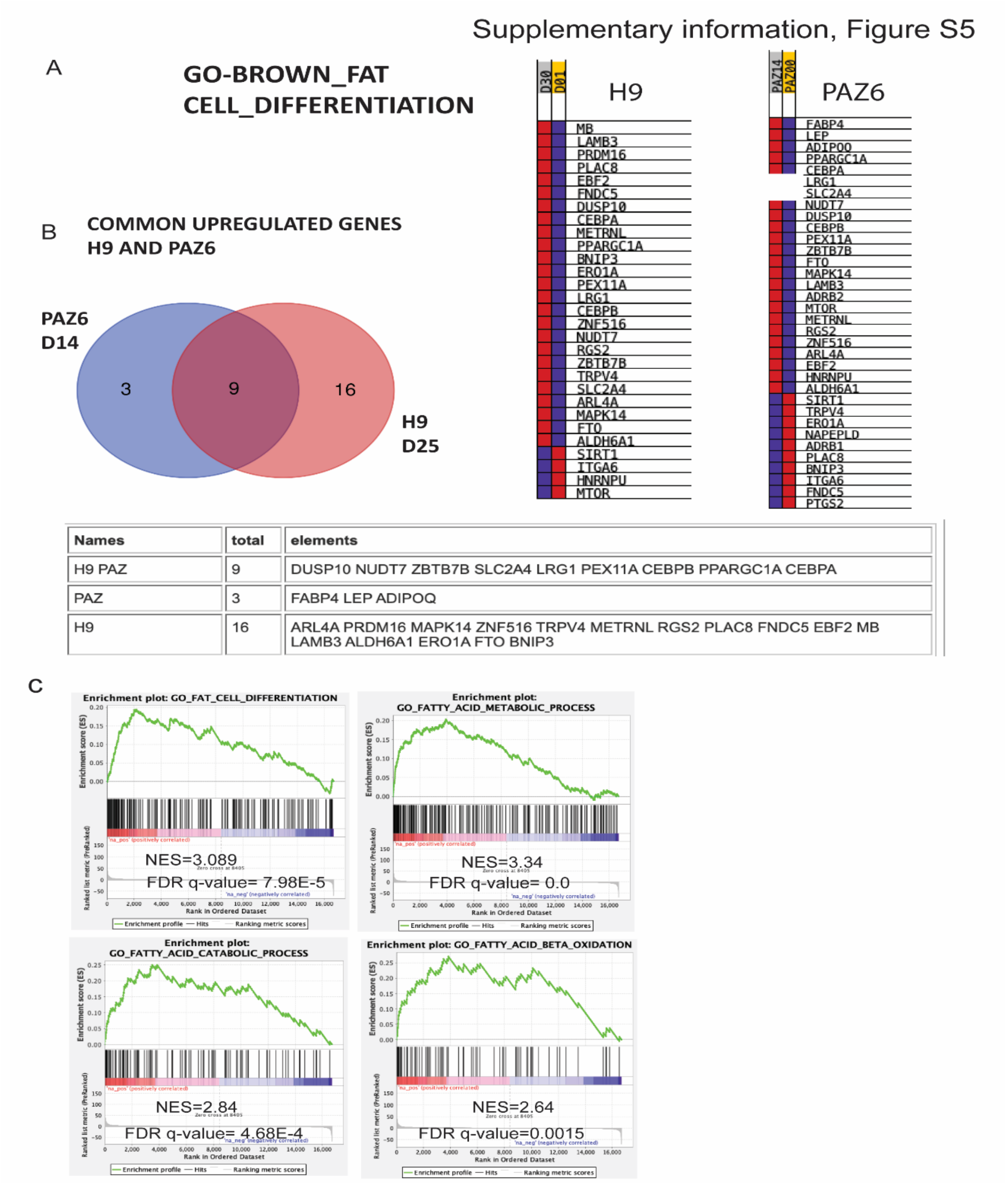

Names	total	elements
H9 PAZ	9	DUSP10 NUDT7 ZBTB7B SLC2A4 LRG1 PEX11A CEBPB PPARGC1A CEBPA
PAZ	3	FABP4 LEP ADIPOQ
H9	16	ARL4A PRDM16 MAPK14 ZNF516 TRPV4 METRNL RGS2 PLAC8 FNDC5 EBF2 MB LAMB3 ALDH6A1 ERO1A FTO BNIP3

Figure S5. Common upregulated genes shared between terminally differentiated human PSC-derived and PAZ6 brown adipocytes and GSEA analysis of mature PAZ6 cells. Related to Figure 4.

(A) Detailed heatmaps showing the gene expression of H9 at D25 and PAZ6 at D14 of differentiation for the dataset GO_Brown_fat_cell_cell differentiation.

(B) Common upregulated genes of H9 at D25 and PAZ6 at D14 of differentiation in the context of GO_Brown_fat_cell_cell differentiation.dataset.

(C) Gene Set Enrichment Analysis of PAZ6 human brown adipocytes at D14 *vs* D0 with GO datasets (“fat cell differentiation” GO:0045444, “fatty acid metabolic process” GO:0006631, “fatty acid catabolic process” GO:0006631 and “fatty acid beta oxidation GO:0006635), (n = 3 independent experiments).

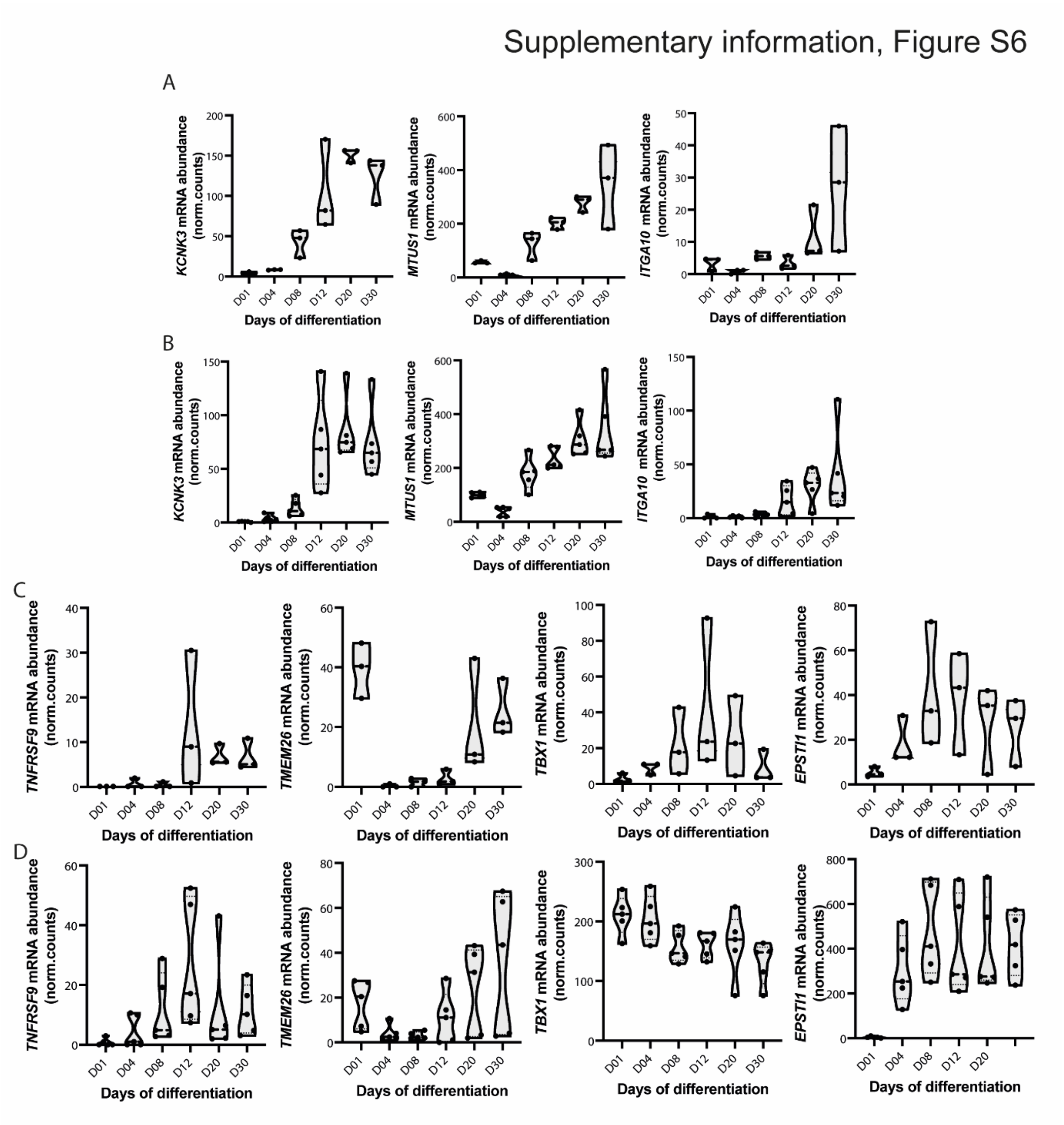

Fig.S6 Stem cell-derived brown adipocytes express known human brown markers. Related to Figure 2.

(A-D) mRNA abundance of the indicated transcripts in H9- (A,C) and KOLF2-C1-derived brown adipocytes (B,D) at the indicated time points. Values are shown as normalised counts and have been extracted from the RNAseq analyses. N= 3 (H9) and 5 (KOLF2-C1) independent experiments.

Supplementary information, Figure S7

A

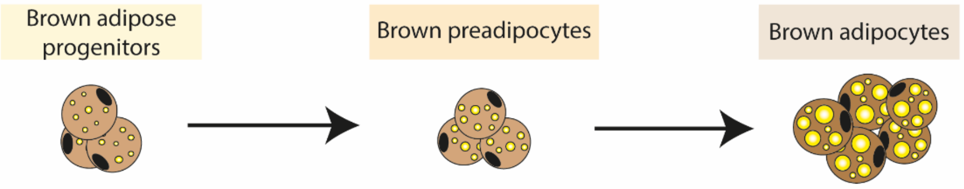

TRANSCRIPTIONAL REGULATION	CEBPA, CEBPB, PPARA PRDM16, AUTS2	EBF1, EBF2, NUPR1	CIDEC, PPARG, PPARGC1A		
SIGNALLING	PIK3R1, IGF1, NDRG1, PRKAR1B, KCNK3				
ADRENERGIC RECEPTORS	ADRB3			ADRB1	
LIPOLYSIS	LIPE		MGLL		
FATTY ACIDS OXYDATION		ACADVL, ACADS, ACSL5, ACSF2	ACAT1, FABP3	ECH1	
ELECTRON TRANSPORT	GLRX5, SUCLG1		SDHA, NDUFV1	ECSIT, CYP2E1	
MITOCHONDRIAL FUNCTIONS		LETMD1, CRLS1	SOD2	QSOX1	ACO2
LIPID REMODELLING	PANK1, AGPAT3				
RETINOIC ACID	RBP4, ALDH1A1				
GLUCOSE METABOLISM	PCK1, PYGB				
INFLAMMATION	CX3CL1, EDN1, SLIT2, CXCL14, IL1B		PTGDS, RARRES2		
EXTRACELLULAR MATRIX	CCDC80, COL3A1, LAMB3	COL4A2, LAMA4			
OTHERS			ATP2B4, DES, EEF2K, CHST1, SYNE2		

Fig.S7. Temporal expression of human BAT markers and functionally relevant proteins. Related to Figure 5.

(A) Analysis of the first developmental step in which the expression of each of the indicated genes is upregulated based on the RNA-seq data. “Brown adipose progenitors” corresponds to D8, “brown preadipocytes” to D12 and “brown adipocytes” to D30. The data shown correspond to both H9 and KOLF2-C1 cell lines.

qPCR primers list

Primer	Forward Sequence	Reverse Sequence
ADRβ3	CTCGACGGGGCTTCTTGG	GAGGCCAGAGGTTTTCCACA
ADRP1	GAGTCGTCTTCGGGACGCGC	TTGGCAACTGCAATTTGCGGC
CD36	TGGAACAGAGGCTGACAACTT	TTGATTTTGATAGATATGGGATGC
C/EBP-α	GACATCAGCGCCTACATCG	GGCTGTGCTGGAACAGGT
C/EBP-β	CCAGCCCCCTCACTAATAGC	CCCTGCTCTGAGCTGTCG
C/EBP-δ	GGACATAGGAGCGCAAAGAA	GCTTCTCTCGCAGTTTAGTGG
DIO2	CCTCCTCGATGCCTACAAAC	GCTGGCAAAGTCAAGAAGGT
EBF2	AAGACCAACAACGGCACTCA	TTCGCAGCATCGACTACACA
GAPDH	AGCCACATCGCTCAGACAC	GCCCAATACGACCAAATCC
MYF5	CTGCCAGTTCTCACCTTCTGA	AACTCGTCCCCAAATTCACCC
NANOG	ATGCCTCACACGGAGACTGT	CAGGGCTGTCCTGAATAAGC
OCT4	GCTTCAAGAACATGTGTAAGCTG	AGGGTTTCCGTTTGCAT
PDGFRα	CCACCTGAGTGAGATTGTGG	TCTTCAGGAAGTCCAGGTGAA
PAX3	ATTGGCAATGGCCTCTCA	AGGGGAGAGCGCGTAATC
PLIN1	AGGGAAGAAGTTGAAGCTTGAGG	TTCTGGAAGCATTCGCAGGT
PPARα	GCACTGGAACTGGATGACAG	TTTAGAAGGCCAGGACGATCT
PPARγ	CGTGGCCGCAGATTTGAAAG	CACGGAGCTGATCCCAAAGT
PRDM16	TGGCTGCTTCTGGACTCA	ATATTATTTACAACGTCACCGTCACT
SOX2	GGGGGAATGGACCTTGTATAG	GCAAAGCTCCTACCGTACCA
TBOX	GCTGTGACAGGTACCCAACC	CATGCAGGTGAGTTGTCAGAA
UCP1	CTCACCGCAGGGAAAGAA	GGTTGCCCAATGAATACTGC
ZIC1	ATCCACAAAAGGACGCACAC	GTCACAGCCCTCAAACTCG

Table S1. Composition Chemically Defined Medium (CDM)

Compounds	Total volume/quantity (concentration)
F-12 Nut Mix (lnvitrogen 31765068)	250ml (50%)
IMDM (lnvitrogen 21980065)	250ml (50%)
HyClone BSA (GE Healthcare SH30574-02)	2.5g (0.Smg/ml)
CD Lipid Concentrate (lnvitrogen 11905031)	5ml (1%)
Insulin (Roche 1376497).Reconstituted in water at l0mg/ml	350µ1 (7µg/ml)
Transferrin (Roche Sigma 10652202001) 30mg/ml	250µ1 (15µg/ml)
Mono-Thioglycerol (Sigma M6145-25ml) 11.5M	20µ1 (0.5mM)

Table S2. Cell culture medium composition at the different stages of differentiation

Stage of differentiation	Day	Media type	Compound	Final concentration
Mesodermal induction (12 well plate)	0	CDM w/o insulin + BSA (see Table S1 for composition)	Insulin (Sigma I9278)	0.35ng/mL
			Fgf2 (Dr. Marko Hyvönen, Cambridge University, in house)	40ng/mL
			Chiron (Sigma CHIR99021)	8µM
Paraxial mesodermal induction (12 well plate)	2	CDM w/o insulin + BSA(see Table S1 for composition)	Insulin (Sigma I9278)	7ng/mL
			Fgf2(Dr. Marko Hyvönen, Cambridge University, in house)	4ng/mL
			Retinoic acid (Sigma R2625)	1µM
Progenitor induction (12 well plate)	4	CDM w/o insulin + BSA (see Table S1 for composition)	Insulin (Sigma I9278)	7ng/mL
			Fgf2 (Dr. Marko Hyvönen, Cambridge University, in house)	4ng/mL
			Chiron (Sigma CHIR99021)	3µM
			LDN 193189 (Sigma SML0559)	100nM
			Ascorbic acid (Sigma A4403)	10mg/ml
Adipose induction 1 (12 well plate)	6-8	DMEM high glucose (Sigma D6546) / Nutrients HAM F12 (v/v) (Sigma N6658) GlutaMAX (Life Technologies 35050038) (1/100) 5% FBS (Life Technologies 16170078) HEPES 15mM (Sigma H0887)	T3 (Sigma T6397)	1nM
			Dexamethasone (Sigma D4902)	100nM
			IBMX (Sigma I7018)	0.25mM
			Biotin (Sigma B4639)	33µM
			Pantothenate (Santa Cruz SC278919)	17µM
			Insulin (Sigma I9278)	500nM
			Rosiglitazone (Sigma R2408)	5µM
			Ascorbic acid (Sigma A4403)	10mg/ml
Adipose induction 2 (12 well plate)	10	DMEM high glucose (Sigma D6546) / Nutrients HAM F12 (v/v) (Sigma N6658)	T3 (Sigma T6397)	1nM
			Dexamethasone (Sigma D4902)	100nM
			Biotin (Sigma B4639)	33µM

		GlutaMAX (Life Technologies 35050038) (1/100)<br>5% FBS (Life Technologies 16170078)<br>HEPES 15mM (Sigma H0887)	Pantothenate (Santa Cruz SC278919)	17µM
			Insulin (Sigma I9278)	500nM
			Rosiglitasone (Sigma R2408)	5µM
Adipose maintenance (12 well plate)	12 – 25	DMEM high glucose (Sigma D6546) / Nutrients HAM F12 (v/v) (Sigma N6658)<br>GlutaMAX (Life Technologies 35050038) (1/100)<br>5% FBS (Life Technologies 16170078)<br>HEPES 15mM (Sigma H0887)	T3 (Sigma T6397)	1nM
			Dexamethasone (Sigma D4902)	100nM
			Biotin (Sigma B4639)	33µM
			Pantothenate (Santa Cruz SC278919)	17µM
			Insulin (Sigma I9278)	500nM
			Rosiglitasone (Sigma R2408)	5µM
			Oleate (Sigma O1008)	100µM

Table S3. Antibodies and dyes list

Antibody/Dye	Supplier	Identifier
AKT	CST	9272S
C/EBPα	Santa Cruz	14AA
COXII (MTCO2 12C4F12)	Invitrogen	A-6404
DIO2	Abcam	ab77779
IRS1	CST	2382S
Ki-67 (8D5)	CST	9449
LipidTOX Deep Red neutral lipid stain	Thermofisher Scientific	H34477
LipidTOX Green neutral lipid stain	Thermofisher Scientific	H34475
LipidTOX red neutral lipid stain	Thermofisher Scientific	H34476
Mitotracker™ Red CMXRos	Thermofisher Scientific	M7512
Anti-mouse IgG, HRP-linked Antibody	CST	7076S
MYF5	Santa Cruz	sc302
p-AKT	CST	4051S
p-IRS1	CST	3070S
p-P70S6K	CST	9206S
P70S6K	CST	2708T
PAX3	DSHB	AB528426
PDGFRα (D13C6) XP	CST	5241
PGC1α	Abcam	ab54481
PLIN1	Progen	GP29
PPARα (H98)	Santa Cruz	sc-9000
PPARγ (E-8)	Santa Cruz	sc-7273
PRDM16	Abcam	ab106410
Anti-rabbit IgG, HRP-linked Antibody	CST	7074S
ß-actin	Abcam	ab16039
TBOX	R&D	AF2085
UCP1	Abcam	ab155117
UCP1	Sigma	U6382
ZIC1	Abcam	134951
ADRP1	Abcam	ab108323

Supplemental Experimental Procedures

Cell culture and maintenance

Two hPSCs lines were employed. The hESC line H9 (WA09, WiCell, Madison, WI) was maintained in Essential 8™ (E8) medium (Gibco) on Vitronectin XF™ (Stemcell technologies) coated (1:1000 dilution) tissue culture-treated 10 cm^2 dishes and passaged mechanically using PBS-EDTA. The hiPSC line KOLF2-C1, a subclone of the hiPSC KOLF2 cell line (HPSI0114i-kolf_2, Human Induced Pluripotent Stem Cell Initiative (HipSCi), http://www.hipsci.org) were maintained in TeSR™-E8™ (Stemcell Technologies) tissue culture-treated 10 cm^2 dishes on Synthemax® II-SC Substrate (10µg/mL) (Sigma- Aldrich) and passaged mechanically using PBS-EDTA or Gentle Cell Dissociation Reagent (Stemcell Technologies). The human immortalised brown adipocyte cell line Paz6 was cultured as described previously (Zilberfarb et al., 1997). Mouse adipocytes were cultured as previously described (Garcia-Casarrubios et al., 2016).

Cell differentiation

For differentiation, pluripotent cells were plated into Matrigel-coated 12-well plates and induced when 70% confluent. At days 0-4, chemically defined medium (CDM; BSA and insulin-free, prepared by Cellular Generation and Phenotyping (CGaP), Wellcome Sanger Institute (Table S1), Hinxton UK was used. From days 6-30, complete medium (DMEM-F12 Ham) (see Table.S2) was used. For functional analyses, cells were plated onto Matrigel-coated glass-bottomed 96-well plates (Eppendorf) at D4 of differentiation; cells were detached using TrypLE (Gibco) and plated at single-cell suspension (one 12-well plate for two 96-well plates).

RNA extraction

Cells were washed three times with PBS and lysed in RLT buffer containing 1% β-ME. Cell lysates were passed five times through a 23G needle before proceeding to RNA extraction using RNeasy Qiagen Kit (Qiagen) following the manufacturer's instructions. RNA concentration was quantified using an Epoch™ 2 microplate reader.

Realtime qPCR

RT-qPCR assessed mRNA levels of genes of interest. cDNA was generated from 500ng isolated RNA using M-MLV reverse transcriptase (Promega) and diluted 1:5 for use in 12µL qPCR reactions using SYBR® Green PCR master mix (Applied Bioscience) run on the Applied Biosystems StepOnePlus™ system Applied Biosystem, Carlsbad, California). Expression values are normalised to GAPDH (See primers Table).

Total RNA library construction and RNAseq

RNA samples were quantified with QuantiFluor RNA System, 1ml from Promega UK Ltd using Mosquito LV liquid platform, Bravo WS and BMG FLUOstar Omega plate reader, and cherrypicked to 100ng / 50µl using Tecan liquid handling platform.

Library construction (poly(A) pulldown, fragmentation, 1st, and 2nd strand synthesis, end prep, and ligation) was carried out using 'NEB Ultra II RNA custom kit' on an Agilent Bravo WS automation system.

PCR was set-up using KapaHiFi Hot start mix, and unique dual indexed tag barcodes on the Agilent Bravo WS automation system. Post PCR, the plate was purified using Agencourt AMPure XP SPRI beads on Caliper Zephyr liquid handling platform. Libraries were quantified with Biotium Accuclear Ultra-high sensitivity dsDNA Quantitative kit using Mosquito LV liquid handling platform, Bravo WS and BMG FLUOstar Omega plate reader. Libraries pooled in equimolar amounts on a Beckman BioMek NX-8 liquid handling platform and pooled libraries were quantified on an

Agilent bioanalyser. Libraries were normalised to 2.8nM. Samples were sequenced on a HiSeq 2500 platform.

RNAseq data analysis

Transcriptomic comparison with publicly available datasets of human and murine adipocytes address (GSE122780, GSE150119) was performed in R using also the ‘DESeq2’ package. In order to the impact of batch effects between the different datasets, Bioconductor’s R function “COMBAT” (Zhang et al., 2018) from package “sva” has been used. The correlation matrices are based on the Pearson’s pairwise correlation between the columns of the batch-corrected datasets. Boxplot distances are based on the Euclidean distances between the columns of the batch-corrected datasets.

Analyses of the transcriptional regulators

The inference of the upstream transcriptional regulators was performed with VIPER (Virtual Inference of Protein-activity by Enriched Regulon analysis) (Alvarez et al., 2016). The algorithm provides a Normalised Enrichment Score (NES), which determines the activation/inhibition status of the transcription factor of interest, based on the observed differential expression of their known gene targets. The TF – target gene interactions network was obtained by DoRothEA (Garcia-Alonso et al., 2019). The outcome of VIPER analysis is a positive NES for activated and negative NES for inhibited TFs, respectively. Only transcription factors with a p-value lower than 0.05 were considered as statistically significant. RNA-seq data for H9-derived myotubes were obtained from the public repository GEO (GSE121154) and analysed as above.

GSEA analysis

Gene Set Enrichment Analysis (GSEA; www.broadinstitute.org/GSEA) was conducted on pre-ranked and non-pre-ranked lists of genes. The ranking was computed

according to the differential transcriptome analysis mentioned above. In particular, we calculated the $-\log_{10}$ of the adjusted p-value referring to the Wald test used for determining the significance of the differential transcriptome expression and we assigned to this value the sign of the fold change. The GSEA analysis was performed using 1000 gene set permutations, and no collapsing. Gene set sizes were selected to be 15-500, classic enrichment and meandiv normalisation mode. The databases used for analyses were: C5.all.V7.0 for D4 and D25 *vs* D0 is and C2.all.V7.0 for D12 *vs* D0. GSEA analysis of CPM non pre-ranked list were processed with the following parameters: the C5.bp.v7.0 database was used with 1000 gene set permutations, and no collapsing. Gene set sizes were selected to be 15-500, Signal2noise metrics for ranking genes, classic enrichment, and meandiv normalisation mode.

Immunocytochemistry

For immunocytochemistry, cells were fixed in 4% PFA for 15 min at room temperature and blocked using 3% FFA-free bovine serum albumin (BSA) 0.1 % Triton X100 or saponin in PBS. Primary antibodies (Table S4) were diluted in PBS, 0.01 % Triton X100 or saponin, 1% FFA-free BSA. Secondary antibodies coupled to Alexa Fluor 488 and 546 or 627 (Raised in Donkey, Thermofisher Scientific) were diluted to 1/1000 in PBS, 0.01% Triton X100 or saponin, 1% FFA-free BSA. Adipocytes were incubated for 45min with HCS LipidTOX Neutral Green / Red or Deep Red neutral lipid stain (Thermofisher Scientific). After a 5min incubation with DAPI (SIGMA, 1/10000), cells were mounted in Fluoromount-G (Southern Biotech) (Table S4). Cell preparations were observed with a confocal microscope (Zeiss LSM 700). Immunocytochemical quantification was performed with the ICY image analysis software (http://icy.bioimageanalysis.org/).

Immunoblot

Cells were lysed in cold RIPA (50mM Tris HCl pH 8,150 mM NaCl ,1% NP-40, 0.5% sodium Deoxycholate, 0.1% SDS,1mM EDTA pH 7.4) buffer and homogenised by vortexing and running samples 5 times through a 23G needle. Samples were spun at maximum speed for 15 min at 4°C, and the supernatant collected. Protein was quantified using BIO-RAD DC™ protein assay (Biorad) following the manufacturer's instructions. Samples of 20µg protein in RIPA and 1X loading buffer (10% β-Mercaptoethanol) were denatured at 95°C for 5min and run on NuPAGE™ 4-12% Bis-Tris Midi Protein Gels (Invitrogen) at 200V for 1h. Protein was transferred to PVDF membranes (Invitrogen) using iBlot™ dry transfer (Thermofisher Scientific). To block unspecific binding of antibodies, membranes were incubated in PBS-T 5% milk for 1h at RT. Primary antibodies were diluted in PBS-T 3% BSA (see Table.S3), and secondary antibodies were diluted in PBS-T 5% milk (see Table.S3).

Insulin sensitivity

Cells were treated with 100nM of insulin for 10 min, at day 25-30 of differentiation, after an o/n incubation in complete medium without serum. The insulin sensitivity of the cells was assessed by measuring the levels of p-AKT, tot AKT, p-IRS1, tot-IRS1, p-P70S6K, and tot-P70S6K. β-ACTIN was the loading control.

Image quantification

Image visualisation was performed using Fiji software (Schindelin et al., 2012). Fluorescent images were analysed using CellProfiler (McQuin et al., 2018) 3.1.9 using custom-built pipelines. Nuclei were identified using the Otsu two-class adaptive thresholding method on the DAPI image channel. Nuclei touching the border of the image were discarded. Subsequently, a cytoplasm mask was created by expanding the nuclei objects by 10 µm. After the exclusion of the nuclei from the cytoplasm, intensity features of the target channel were measured inside the cytoplasm area. The

percentage of positive cells was calculated by means of supervised machine learning using the classifier tool in CellProfiler Analyst 2.2.1 (Jones et al., 2008). The RandomForest classifier was applied to the intensity features of the target channel to create a binary classifier. All trained classifiers had a classification accuracy of at least 80%. Finally, the percentage of positive cells were scored using this classifier accordingly.

Seahorse oxygen consumption measurements

Cells were differentiated in 24-well Seahorse V17 culture plate for 25 days. Before oxygen consumption rate (OCR) assay, complete medium was replaced with Seahorse medium without serum and cytokines. With a Seahorse XF24 analyser, OCR was measured with small molecule inhibitors added through the injection ports. The following concentrations of activators and inhibitors were used: 100, 10 and 1µM mirabegron, 2µM oligomycin, 5µM FCCP and 1µM each of antimycin A and rotenone. Basal, uncoupled, and maximal respiration rates were calculated upon the subtraction of the non-mitochondrial oxygen consumption obtained at the end of each assay by the addition of antimycin A and rotenone. The values obtained were normalised to total mg DNA per well as measured by Quant-iT™ PicoGreen™ dsDNA Assay Kit (Invitrogen). For mouse adipocytes, the following concentrations of activators and inhibitors were used: 1µM oligomycin, 0.9µM FCCP, and 1µM each of antimycin A and rotenone.

Glucose uptake

Norepinephrine (NE)-induced-glucose uptake was assayed according to the established protocol from a commercial glucose uptake kit (Abcam). In brief, at day 25-30 of differentiation, human PSC-derived BAs seeded in 12-well plates were fasted overnight in complete medium without serum. The next day cells were treated with

vehicle or indicated concentration of NE. After 2h of incubation, cells were washed three times with cold PBS and lysed with extraction buffer, frozen at −80 °C for 10 min and heated at 85 °C for 40 min. After cooling on ice for 5 min, the lysates were neutralised by adding neutralisation buffer and centrifuged. The remaining lysate was then diluted with assay buffer. Finally, the colorimetric end product generation was set up by two amplification steps according to the manufacturer's instructions in the kit and then detected at 412 nm using a Spark microplate reader (Tecan).

cAMP measurements

The cAMP assay was performed using the cAMP ParameterAssay Kit (R&D Systems, Minneapolis, MN, USA). In brief, at day 25-30 of differentiation, hPSC-derived BAs seeded in 12-well plates were fasted overnight in complete medium without serum. The next day cells were treated with vehicle or indicated concentration of NE, mirabegron, isoproterenol, and forskolin for 2h. Cells were washed three times in cold PBS, resuspended in cell lysis buffer 5 (diluted 1:5)*, and frozen at ≤ -20 °C. Then the cells were thawed with gentle mixing. The freeze/thaw cycle was repeated once. Samples were then spun at 600 x g for 10 min at 2-8 °C to remove cellular debris, and the supernatant was stored at ≤ -20 °C. The assay was carried out following the manufacturer instructions (R&D Systems, Minneapolis, MN, USA).

Alvarez, M.J., Shen, Y., Giorgi, F.M., Lachmann, A., Ding, B.B., Ye, B.H., and Califano, A. (2016). Functional characterization of somatic mutations in cancer using network-based inference of protein activity. Nat Genet *48*, 838-+.

Garcia-Alonso, L., Holland, C.H., Ibrahim, M.M., Turei, D., and Saez-Rodriguez, J. (2019). Benchmark and integration of resources for the estimation of human transcription factor activities. Genome Res *29*, 1363-1375.

Garcia-Casarrubios, E., de Moura, C., Arroba, A.I., Pescador, N., Calderon-Dominguez, M., Garcia, L., Herrero, L., Serra, D., Cadenas, S., Reis, F., *et al.* (2016). Rapamycin negatively impacts insulin signaling, glucose uptake and uncoupling protein-1 in brown adipocytes. Biochim Biophys Acta *1861*, 1929-1941.

Jones, T.R., Kang, I.H., Wheeler, D.B., Lindquist, R.A., Papallo, A., Sabatini, D.M., Golland, P., and Carpenter, A.E. (2008). CellProfiler Analyst: data exploration and analysis software for complex image-based screens. BMC Bioinformatics *9*, 482.

McQuin, C., Goodman, A., Chernyshev, V., Kamentsky, L., Cimini, B.A., Karhohs, K.W., Doan, M., Ding, L., Rafelski, S.M., Thirstrup, D., *et al.* (2018). CellProfiler 3.0: Next-generation image processing for biology. PLoS Biol *16*, e2005970.
Schindelin, J., Arganda-Carreras, I., Frise, E., Kaynig, V., Longair, M., Pietzsch, T., Preibisch, S., Rueden, C., Saalfeld, S., Schmid, B., *et al.* (2012). Fiji: an open-source platform for biological-image analysis. Nat Methods *9*, 676-682.
Zhang, Y., Jenkins, D.F., Manimaran, S., and Johnson, W.E. (2018). Alternative empirical Bayes models for adjusting for batch effects in genomic studies. BMC Bioinformatics *19*, 262.
Zilberfarb, V., Pietri-Rouxel, F., Jockers, R., Krief, S., Delouis, C., Issad, T., and Strosberg, A.D. (1997). Human immortalized brown adipocytes express functional beta3-adrenoceptor coupled to lipolysis. J Cell Sci *110 (Pt 7)*, 801-807.
